# Delineating the Molecular Basis of the Calmodulin–bMunc13-2 Interaction by Cross-Linking/Mass Spectrometry—Evidence for a Novel CaM Binding Motif in bMunc13-2

**DOI:** 10.3390/cells9010136

**Published:** 2020-01-07

**Authors:** Christine Piotrowski, Rocco Moretti, Christian H. Ihling, André Haedicke, Thomas Liepold, Noa Lipstein, Jens Meiler, Olaf Jahn, Andrea Sinz

**Affiliations:** 1 Department of Pharmaceutical Chemistry and Bioanalytics, Institute of Pharmacy, Charles Tanford Protein Center, Martin Luther University Halle-Wittenberg, D-06120 Halle/Saale, Germany; 2 Center for Structural Biology, Department of Chemistry, Vanderbilt University, Nashville, TN 37221, USA; 3 Biophysical Chemistry, Institute of Chemistry, Martin Luther University Halle-Wittenberg, D-06120 Halle/Saale, Germany; 4 Proteomics Group, Max Planck Institute of Experimental Medicine, D-37075 Göttingen, Germany; 5 Department of Molecular Neurobiology, Max Planck Institute of Experimental Medicine, D-37075 Göttingen, Germany

**Keywords:** Munc13, calmodulin, cross-linking, mass spectrometry, protein*–*protein interaction

## Abstract

Exploring the interactions between the Ca^2+^ binding protein calmodulin (CaM) and its target proteins remains a challenging task. Members of the Munc13 protein family play an essential role in short-term synaptic plasticity, modulated via the interaction with CaM at the presynaptic compartment. In this study, we focus on the bMunc13-2 isoform expressed in the brain, as strong changes in synaptic transmission were observed upon its mutagenesis or deletion. The CaM–bMunc13-2 interaction was previously characterized at the molecular level using short bMunc13-2-derived peptides only, revealing a classical 1–5–10 CaM binding motif. Using larger protein constructs, we have now identified for the first time a novel and unique CaM binding site in bMunc13-2 that contains an *N*-terminal extension of a classical 1–5–10 CaM binding motif. We characterize this motif using a range of biochemical and biophysical methods and highlight its importance for the CaM–bMunc13-2 interaction.

## 1. Introduction

Ca^2+^ acts as a key regulator of multiple cellular processes, such as cell proliferation, differentiation, apoptosis, and neurotransmitter release [1,2]. Changes in Ca^2+^ concentrations are sensed by various proteins that either bind to Ca^2+^ itself or interact with Ca^2+^ binding proteins. A prominent Ca^2+^ binding protein is calmodulin (CaM), a small 17-kDa acidic protein that is highly conserved in all eukaryotes and has an identical amino acid sequence in vertebrates [3,4]. CaM is a 148-amino acid protein arranged in two globular domains, each containing two Ca^2+^ binding EF-hands that are connected by a flexible linker [5,6,7]. CaM evolved to enable promiscuous target binding, requiring few specific interactions to interact with its more than 300 known target proteins [8,9,10]. Target binding occurs through a structural rearrangement of CaM, allowing hydrophobic amino acids of CaM to arrange into a binding pocket [11]. In Ca^2+^-dependent binding, targets adopt a basic amphiphilic helical structure where the hydrophobic anchor amino acids at specific positions define different Ca^2+^ binding motifs, such as 1–8–14 or 1–5–10 motifs [12]. Ca^2+^-independent IQ-motifs also feature a helical structure with a positive net charge, but with no or only low amphiphilicity [13]. Known CaM binding motifs are summarized in CaM target databases that aid in determining CaM interaction sites and in identifying novel CaM targets [14,15].

**Figure 1:**
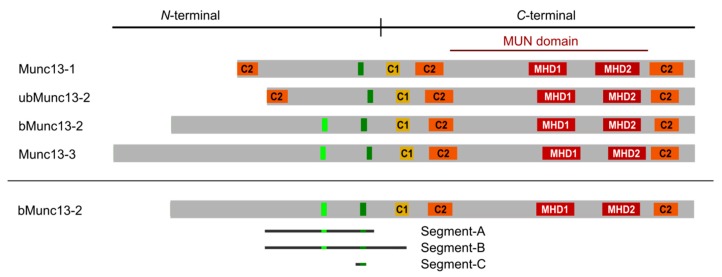
Domain structure of Munc13 proteins. All Munc13 isoforms share a conserved *C*-terminal priming unit and a central regulatory unit. Munc13-1 and ubMunc13-2 have a homologous *N*-terminal region containing an additional C2 domain. CaM-binding sites (dark green bars) are located in the regulatory unit. In bMunc13-2 and Munc13-3, another candidate CaM binding site (light green bars) was initially identified by computational methods, but found to be deficient of CaM-binding in the protein context [28]. C1 = diacylglycerol binding domain (yellow), C2 = Ca^2+^-binding/protein interaction domain (orange), MHD = Munc homology domain (red). The bMunc13-2 segments investigated in this study are displayed. Segment-A (aa 367–780), segment-B (aa 367–903), and segment-C (aa 703/704–742).

At the presynaptic compartment, where synaptic vesicles (SVs) fuse with the plasma membrane to release neurotransmitter into the synaptic cleft, regulation of protein function by CaM binding plays a major role, with over 75 proteins proposed as CaM targets [16]. One such well-studied regulatory interaction is the Ca^2+^-dependent CaM/Munc13 interaction, which occurs after activity-induced Ca^2+^ influx. Munc13s are absolutely essential for the assembly of SNARE complexes [17,18], which link the SV membrane with the active zone plasma membrane [19]. This generates a pool of readily releasable SVs, where the molecular machinery is assembled in a form that allows a local and accurate neurotransmitter release in response to an action potential-evoked Ca^2+^-influx. The Munc13 protein family is composed of multiple members, four of which are expressed in the central nervous system (CNS) [20,21]. Munc13-1 is the dominant isoform expressed in the vast majority of CNS synapses [22], with some synapse types expressing additional isoforms. Hippocampal synapses co-express bMunc13-2 as the major Munc13-2 isoform [23]. It is derived as a splice isoform from the *Unc13B* gene, together with ubMunc13-2. In contrast, cerebellar synapses co-express primarily Munc13-3 with Munc13-1 [24]. All Munc13 isoforms share a conserved *C*-terminal domain structure composed of a C1 domain, a central C2 domain, a MUN domain, and a *C*-terminal C2 domain (Figure 1). Interestingly, the *N*-terminal domains are rather divergent and contain large unstructured regions, for which little functional information is available to date.

In line with the essential role of Munc13s in generating fusible SVs, Munc13-1 has recently been implicated in the determination of synaptic strength [25]. The role of Munc13-2 proteins, however, is still not fully understood. Evidence points to an involvement in the fine-tuning of synaptic efficacy and plasticity [26,27,28,29]. A major pathway that regulates synaptic plasticity via Munc13 proteins is the Ca^2+^/CaM-dependent pathway. We have previously shown that all Munc13 isoforms contain a single, functional CaM binding site at a similar *N*-terminal position of their C1 domains [28] and that Ca^2+^/CaM binding to that site increases synaptic efficacy during activity. In bMunc13-2 and Munc13-3, another candidate CaM binding site was predicted and indeed shown to bind CaM at the level of peptides [28]. However, cosedimentation experiments with GST-bMunc13-2(367–780) and mutated variants thereof provided strong evidence that CaM binds in a 1:1 stoichiometry and that the further *C*-terminally located CaM binding site is the relevant one in the protein context [28]. The CaM/Munc13-1 interaction was studied in great detail at the functional level both in vitro [30] and in vivo [31], and at the structural level [32]. In particular, the canonical 1–5–8 CaM binding sites of the closely related isoforms Munc13-1 and ubMunc13-2 were shown to extend *C*-terminally, leading to the identification of a secondary hydrophobic contact point with CaM and thus to the definition of a novel 1–5–8–26 CaM binding motif [32]. Evidence from NMR experiments indicated that the elongated motif may mediate sequential CaM binding, as a potential mechanism for Ca^2+^-sensing over a broad concentration range. However, no such motif is present in bMunc13-2, raising the question of whether other elongated CaM binding sites exist in this isoform, particularly in view of the fact that bMunc13-2 exhibits the strongest CaM-dependent effects on synaptic plasticity in cultured hippocampal neurons [28].

We study the CaM/bMunc13-2 interaction in more detail by using larger bMunc13-2 constructs and elongated peptides, rather than minimal CaM-binding peptides. We applied an integrated approach based on chemical cross-linking/mass spectrometry (XL-MS), a method to investigate protein conformations and map protein–protein interactions [33,34,35,36,37,38] by covalently connecting functional groups of amino acid side chains that are in spatial proximity to a protein or a protein complex. Depending on the specific spacer length of the cross-linking reagents, mass spectrometric analysis reveals distance information for the covalently connected amino acids, which can be used to derive three-dimensional protein structures by computational modeling approaches [39,40,41]. We used XL-MS and complementary interaction analyses to show that the CaM binding to bMunc13-2 involves the *N*-terminal elongation of the previously defined CaM binding site. This elongated CaM-binding sequence is unique in bMunc13-2 and confers a substantially higher affinity for CaM binding. Our data underscore the importance of considering extended sequence motifs including core CaM binding domains and their flanking regions for a deeper understanding of CaM-dependent regulation of Munc13 proteins in the presynapse.

## 2. Materials and Methods

### 2.1. Materials

CaM (bovine brain) was obtained from Athens Research (Athens, GA, USA). Cross-linkers were purchased from Thermo Fisher Scientific (Dreieich, Germany) (s-GMBS, BS^3^ and photo-Met) or synthesized in house (DSBU) [42]. The proteases trypsin (porcine) and GluC were obtained from Promega GmbH (Mannheim, Germany). Chemicals were obtained from Sigma Aldrich (Taufkirchen, Germany), Merck (Darmstadt, Germany), and VWR (Darmstadt, Germany). Prestained Protein Ladder was purchased from Thermo Fisher Scientific. For HPLC, solvents were LC-MS grade (VWR, Darmstadt, Germany). MilliQ water was produced by a TKA X-CAD system (Thermo Fisher Scientific, Niederelbert, Germany).

### 2.2. Expression and Purification of Recombinant CaM and bMunc13-2 Segments

Photo-Met labeled CaM (Figure S1) was expressed and purified as described previously [43]. Recombinant photo-Met-labeled CaM was stored at −20 °C in 20 mM HEPES, 150 mM NaCl buffer (pH 7.2 at 25 °C). Recombinant rat bMunc13-2 segments (segment-A, aa 367–780; segment-B, aa 367–903; Figure S2) were expressed with *N*-terminal glutathione-S-transferase (GST) tag in the *E. coli* strain BL21(DE3) c^+^RIL pGEX-4T-1. The *E. coli* strain was grown on TB medium to an OD_600nm_ of 0.6 at 37 °C. Protein production was induced by adding IPTG to a final concentration of 0.1 mM. Cells were further grown at 18 °C and harvested after incubation overnight. GST-tagged segments were enriched from cell extracts by GST affinity chromatography and strong cation exchange chromatography on an ÄKTA FPLC system (GE Healthcare Life Sciences, Freiburg, Germany). The GST tag was cleaved by thrombin (0.08 mg/mL protein solution) overnight at 6 °C. Purified segments were stored at −20 °C in 20 mM HEPES, 300 mM NaCl, 0.5 mM TCEP, 10% glycerol buffer (pH 7.2 at 25 °C). Segment-C and its variants were synthesized by standard solid phase peptide synthesis using fluorenylmethyloxycarbonyl (Fmoc) chemistry.

### 2.3. (Photo-)Chemical Cross-Linking

Cross-linking experiments were performed at 25 °C with free Ca^2+^ concentrations ranging between 75 nm and 1 mM. Ca^2+^ levels were controlled using an EGTA chelating system [44].

*Photo-cross-linking between photo-Met-labeled CaM and bMunc13-2 segments.* Photo-cross-linking reactions were conducted in 20 mM HEPES, 250 mM NaCl, 0.5 mM TCEP, 7.5% glycerol buffer (pH 7.2 at 25 °C). Photo-Met labeled CaM (10 µM) was incubated in the respective Ca^2+^/EGTA buffer for 15 min at 25 °C. Afterwards, 10 µM segment-A, -B, or -C were added and the mixture was incubated for 60 min at 25 °C. To induce photo-cross-linking, the reaction mixtures were subjected to UV-A irradiation (360 nm) at 8000 mJ/cm^2^ in a home-built irradiation chamber [45].

*Chemical cross-linking between CaM and bMunc13-2 segments.* All reactions were conducted in 20 mM HEPES, 230 mM NaCl, 0.5 mM TCEP, 7.5% glycerol buffer (pH 7.2 at 25 °C) with CaM (bovine brain). For BS^3^ (D_0_/D_4_) and DSBU cross-linking, CaM (10 µM) was incubated in the respective Ca^2+^/EGTA buffer for 15 min at 25 °C. Afterwards, 10 µM segment-A was added and incubated at 25 °C for 60 min, before the cross-linker was added to a final concentration of 1 mM. The cross-linking reaction was allowed to proceed for 60 min at 25 °C before the reaction was quenched by adding NH_4_HCO_3_ or TRIS to a final concentration of 20 mM.

Cross-linking with s-GMBS was performed as a two-step reaction. In a first step, CaM was labeled with s-GMBS by mixing 60 µM CaM with a 50-fold molar excess of s-GMBS and incubation at 25 °C for 60 min. The reaction was quenched by the addition of NH_4_HCO_3_ (20 mM final concentration) and excess cross-linker was removed by centrifugation with ZebaSpin desalting columns (7 kDa cut-off, Thermo Scientific). After incubating 10 µM s-GMBS-labeled CaM in a Ca^2+^/EGTA buffer for 15 min at 25 °C, 10 µM segment-A was added and the cross-linking reaction of the sulfhydryl-reactive site of s-GMBS was allowed to proceed for 60 min at 25 °C. The reaction was quenched by the addition of glutathione (20 mM final concentration).

### 2.4. Enzymatic Digestion

For in-solution digestion, the protein solution was mixed with a solution containing 8 M urea and 400 mM NH_4_HCO_3_ at 1:3 (*v*/*v*) ratio or 1.5% deoxycholate were added. The proteins were reduced with dithiothreitol (DTT) and alkylated with iodoacetamide. The solution was diluted with water 1:10 (*v*/*v*), followed by the addition of GluC at a protein:enzyme ratio of 30:1 (*w*/*w*) and incubation at 30 °C overnight. Subsequently, trypsin was added at a protein:enzyme ratio of 30:1 (*w*/*w*) and digestion was performed at 37 °C for 4 h. In-gel digestion was performed by excising the bands of interest from the SDS-gel. Gel pieces were subjected to in-gel digestion as described previously using GluC/trypsin or AspN/trypsin as digestion enzymes [45].

### 2.5. Liquid Chromatography/Mass Spectrometry (LC/MS)

Peptide digestion mixtures were separated on an Ultimate 3000 RSLC NanoHPLC system (Thermo Fisher Scientific); precolumn: C8 reversed phase, Acclaim PepMap, 300 µm × 5 mm, 5 µm, 100 Å (Thermo Fisher Scientific) or C18 reversed phase, Acclaim PepMap, 300 µm × 5 mm, 5 µm, 100 Å (Thermo Fisher Scientific); separation column: C18 reversed-phase, Acclaim PepMap, 75 μm × 250 mm, 2 µm, 100 Å (Thermo Fisher Scientific) or PicoFrit C18 nanospray column, 75 µm ID, 10 μm tip (New Objective), packed with ReproSil-Pur 120 C18-AQ, 1.9 µm. Peptide mixtures were washed on the precolumn with water containing 0.1% TFA for 15 min, before the peptides were separated on the separation column using gradients from 1% to 35% (40%) B (90 min), 35% (40%) to 85% B (5 min) followed by 85% B (5 min), with solvent A: 0.1% formic acid (FA) in water and solvent B: 0.08% FA in acetonitrile. The nano-HPLC system was directly coupled to a Fusion Tribrid Orbitrap mass spectrometer equipped with a Nanospray Flex Ion Source (Thermo Fisher Scientific, Bremen, Germany). Data were acquired using the data-dependent MS/MS mode, i.e., each high-resolution full-scan in the Orbitrap (*m*/*z* 300 to 2000, R = 120,000) was followed by high-resolution product ion scans in the orbitrap (R = 15,000) within 5 s, starting with the most intense signal in the full-scan mass spectrum (isolation window 2 Th). Fragmentation was achieved by CID (35% normalized collision energy), HCD (29% normalized collision energy) or stepped HCD (30% ± 3% normalized collision energy). Dynamic exclusion (exclusion duration: 60 s, exclusion window: ±2 ppm) was enabled to allow detection of less abundant ions. Data acquisition was controlled with Xcalibur v3.0.63 (Thermo Fisher Scientific).

### 2.6. Identification of Cross-Links

Data were analyzed with the StavroX (v3.4.9 to v3.6.6) and MeroX (v1.4.12 to v1.6.6) software tools [46,47] (www.StavroX.com). Mass tolerances were 3 ppm (MS) and 10 ppm (MS/MS); the signal-to-noise ratio was set to 2.0. Cross-linking sites were specifically defined for each cross-linking principle (Figure S3): for photo-Met, all 20 proteinogenic amino acids were considered as potential reaction sites; for BS^3^ and DSBU, lysines, serines, threonines, *N*-termini [48] were considered as reaction sites; for s-GMBS, cysteines were included. For photo-Met, oxidation of Met, carbamidomethylation of Cys and exchange of Met with photo-Met were considered as variable modifications. For BS^3^ and DSBU, carbamidomethylation of Cys was set as static modification; for BS^3^, DSBU, and s-GMBS, oxidation of Met was set as variable modification. Three missed cleavage sites were allowed for each cleavage site. All cross-links were manually validated.

### 2.7. Docking of CaM/bMunc13-2 Segment-C Complexes

Details on the modeling process are described in Supplementary Material III (Supplementary Methods: Protocol capture page for modeling approach).

Models of the CaM/segment-C (aa 704–742) interaction were generated using the ROSETTA FlexPepDock ab initio application [49]. All steps were performed with ROSETTA 2015.31.58019 and ROSETTA 2016.32.58837. To enable the docking process, an α-helical 3D-structure of the peptide was created by the BuildPeptide command and manually placed into the CaM structure derived from the PDB-file 2O60 (CaM/NO synthase peptide complex [50]) in PyMOL (v1.2r1). Input for the FlexPepDock application was the structure generated and a constraints file, which contained information about the expected localization of the CaM binding site of bMunc13-2 within CaM obtained from PDB structures 2KDU [32] and 2O60 [50]. The 10,000 models generated were rescored by the distance constraints obtained from the cross-linking experiments. Distance thresholds were set to ≤8 Å for photo-Met and ≤40 Å for DSBU [39]. A KofN selection (the best K crosslinks from N total; K = 5 to 7; N = 10) was applied to allow for only a fraction of all cross-links fitting into one model. Afterwards, the models were clustered by atom_pair_constraints and sorted by the given re-weighted score and atom pair constraints. Models, which were found to satisfy 5, 6 as well as 7 of 10 cross-links, were selected and subjected to relaxation to prevent structural tensions. Subsequently, the five top-scoring models were applied to two additional cycles of rescoring and relaxation. The resulting models were manually inspected and two were identified as the final models.

### 2.8. Native Electrospray Ionization Mass Spectrometry

Native ESI-MS was performed on a high-mass Q-TOF2 mass spectrometer (Waters Micromass/MS Vision, Almere, Netherlands). The 10 µM solutions of CaM and bMunc13-2 segment-A, as well as a mixture of 3.33 µM CaM and 12 µM segment-C, were prepared in 300 mM ammonium acetate (pH 7.0) using centrifugation units (3 or 10 kDa cut-off, Amicon Ultra, 0.5 mL, Millipore, Darmstadt, Germany). Data were acquired using a capillary voltage of 1.38 kV, a cone voltage of 160–180 V, an extraction cone voltage of 5–10 V, and collision energies between 10 and 80 V.

### 2.9. Isothermal Titration Calorimetry

Isothermal titration calorimetry (ITC) measurements were performed at 25 °C using a MicroCal VP-ITC system (MicroCal Inc., Northampton, MA) with 20 mM HEPES, 230 mM NaCl, 0.5 mM TCEP buffer (pH 7.2; 25 °C). All solutions were degassed for 10 min under vacuum prior to measurement. A CaM solution (4 µM) was present in the sample cell, while bMunc13-2 segment-C solution (80 µM) was applied via syringe. For ITC measurements, segment-C was injected in 5-µL steps into the sample cell. As a reference, the heat of dilution was determined by injection of the peptide into the ITC buffer without CaM that was subtracted afterwards from the titration curve. Data analysis was performed by the ITC module implemented in Origin 6.0 (MicroCal Inc., Northampton, MA, USA).

### 2.10. Photoaffinity Labeling-Based Competition Assay

Photoreactive peptides were synthesized by Fmoc chemistry using the amino acid derivative Fmoc-para-benzoylphenylalanine (Bpa, Novabiochem). Photoaffinity labeling (PAL)-based competition experiments with recombinant CaM were performed and analyzed as described previously [28]. Briefly, CaM (5 µM) and segment-C-Bpa (5 µM) or segment-CS-Bpa (5 µM) were incubated in 50 mM HEPES (pH 7.2), 150 mM KCl, 5 mM DTT for 2 h at RT in the dark with (200 µM Ca^2+^) or without (2 mM EGTA) calcium, and in the presence of increasing concentrations (0–250 μM) of competitor peptides. Following UV irradiation, photoadduct formation was visualized and quantified by SDS-PAGE and linear matrix-assisted laser desorption/ionization–time-of-flight–mass spectrometry (MALDI-TOF-MS).

### 2.11. Surface Plasmon Resonance Spectroscopy

The CaM–bMunc13-2 segment-C interaction was studied by surface plasmon resonance (SPR) using an MP-SPR Navi 200 OTSO instrument (BioNavis, Tampere, Finland). All steps were performed with a flow rate of 30 µL/min and sensograms were recorded at a laser wavelength of 670 nm. First, 2 µM of tetrameric streptavidin, reconstituted in 20 mM HEPES pH 7.5, was injected twice to be immobilized on a PEG-biotin sensor chip (Cenibra GmbH, Bramsche, Germany). Photo-Met-labeled CaM was biotinylated in 20 mM HEPES, pH 6.5, with a 10-fold molar excess EZ-Link NHS-LC-biotin (Thermo Fisher Scientific, Dreieich, Germany) over protein concentration. The reaction was performed at 25 °C for 1 h and quenched by the addition of NH_4_HCO_3_ (20 mM final concentration). Afterwards, excess of biotinylation reagent was removed by ZebaSpin desalting columns (7 kDa cut-off) performing a buffer exchange to 20 mM HEPES, pH 7.5, at the same time. The biotinylation was verified by MALDI-TOF-MS (Figure S4). 7 µM of biotinylated CaM was injected to immobilize the CaM onto streptavidin in the sample cell; CaM was collected and reinjected twice to maximize the amount of immobilized protein. Segment-C and its variants were dissolved in 20 mM HEPES, 230 mM NaCl, 0.5 mM TCEP buffer (pH 7.2; 25 °C). The interaction was studied by injection of 0.1–1 µM segment-C variant. K_D_ values were obtained using the TraceDrawer software (Ridgeview, Vänge, Sweden).

## 3. Results

### 3.1. Ca^2+^-Dependent Formation of a Stoichiometric Complex between CaM and bMunc13-2

As bMunc13-2 is a >220 kDa multi-domain protein that is difficult to express, two different constructs and a synthetic peptide, all derived from its *N*-terminal region, were employed as proxies for the intact protein to study the CaM–bMunc13-2 interaction. Segment-A (aa 367–780) contains both the established CaM binding site and the predicted candidate CaM binding site, of which only the former was shown to bind to CaM at the protein level. Segment-B (aa 367–903) contains the sequence of segment-A and is *C*-terminally elongated to include the C1 domain. Segment-C (aa 704–742) spans only the established CaM binding site and the ~15 aa stretch located directly *N*-terminal of it (Figure 1). As a starting point for investigating complex formation, we expressed segment-A with an *N*-terminal GST-tag, a construct successfully used in previous cosedimentation experiments [28]. For our cross-linking studies, the GST-tag was removed via its thrombin cleavage site to yield the native bMunc13-2 segment-A with 12 additional *N*-terminal amino acids originating from the multiple cloning site of the plasmid (Figure S2A).

**Figure 2:**
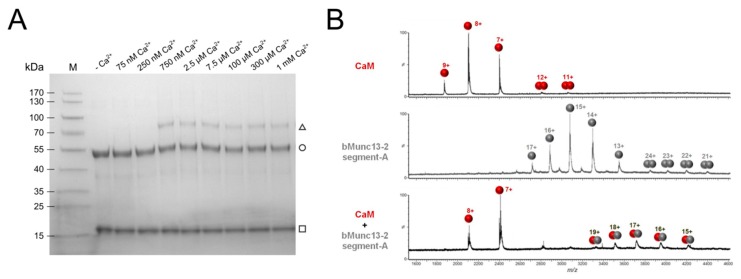
Ca^2+^-dependent complex formation between CaM and bMunc13-2 segment-A. (**A**) Gradient (4-20%) SDS-PAGE gel illustrating cross-linking of the CaM/segment-A complex at various Ca^2+^ concentrations (adjusted by an EGTA buffer system). Covalent fixation of the complex was achieved by cross-linking of photo-Met residues incorporated in CaM. Proteins are visualized by Coomassie staining. (M) Prestained Protein Ladder, (□) CaM, (○) segment-A and (∆) CaM/segment-A complex. (**B**) Native MS mass spectra of CaM alone (upper panel), segment-A alone (middle panel) and a segment-A/CaM complex (lower panel). Proteins (10 µM) were applied in 300 mM ammonium acetate (pH 7) and measurements were performed on a high-mass Q-TOF mass spectrometer. MS data were acquired using a capillary voltage of 1.38 kV, a cone voltage of 180 V, and a collision energy of 60 V. Measurements were performed at residual Ca^2+^ concentration known to be around 5 µM [55].

With the aim of identifying the effective Ca^2+^ concentration range in vitro, we first studied the Ca^2+^ dependence of complex formation between CaM and segment-A by photo-cross-linking experiments with photo-Met-labeled CaM [43]. CaM contains nine methionines (Figure S1) that were all found to be replaced by photo-Met with incorporation rates of up to 30% [43]. The artificial amino acid photo-Met contains a diazirine moiety that is activated upon UV-A irradiation (~360 nm), resulting in the formation of a covalent bond between amino acids in close spatial proximity (Figure S3A) [51]. When we evaluated complex formation between photo-Met-labeled CaM and segment-A at different Ca^2+^ concentrations (Figure 2A), we identified 750 nM Ca^2+^ as a low, but already saturating concentration. For all later experiments aiming at structural characterization of the complex, we chose this low concentration together with a commonly used excessive concentration of 1 mM Ca^2+^ to be able to study Ca^2+^-dependent conformational differences. The apparent molecular weight of ~70 kDa of the complex indicated a 1:1 stoichiometry, which was confirmed by cross-linking-independent approaches for detection of the intact CaM/segment-A complex, such as native ESI-MS measurements (Figure 2B) and native gel electrophoresis (Figure S5). Notably, the observation of a 1:1 stoichiometry was also in agreement with the results of our previous cosedimentation experiments employing GST-bMunc13-2(367–780) [28]. Taken together, our assays demonstrate that CaM and segment-A of bMunc13-2 form a stoichiometric complex at apparent Ca^2+^ concentrations in the high nanomolar range, which reflects the Ca^2+^ levels required for the interaction of full-length ubMunc13-2 and immobilized CaM [52], and overall fits well within the range of presynaptic Ca^2+^ concentrations observed during synaptic activity [53]. We would like to mention, however, that in our experience the readout particularly from cross-linking-based Ca^2+^ titration experiments can be considerably assay-dependent and we thus refrain from further elaborating on the Ca^2+^ sensitivity of the bMunc13-2–CaM interaction by means of exact numbers and potential physiological relevance, but focus on the structural characterization of the bMunc13-2/CaM complex.

### 3.2. Structural Characterization of CaM–bMunc13-2 Segment-A Interaction by XL-MS

**Figure 3:**
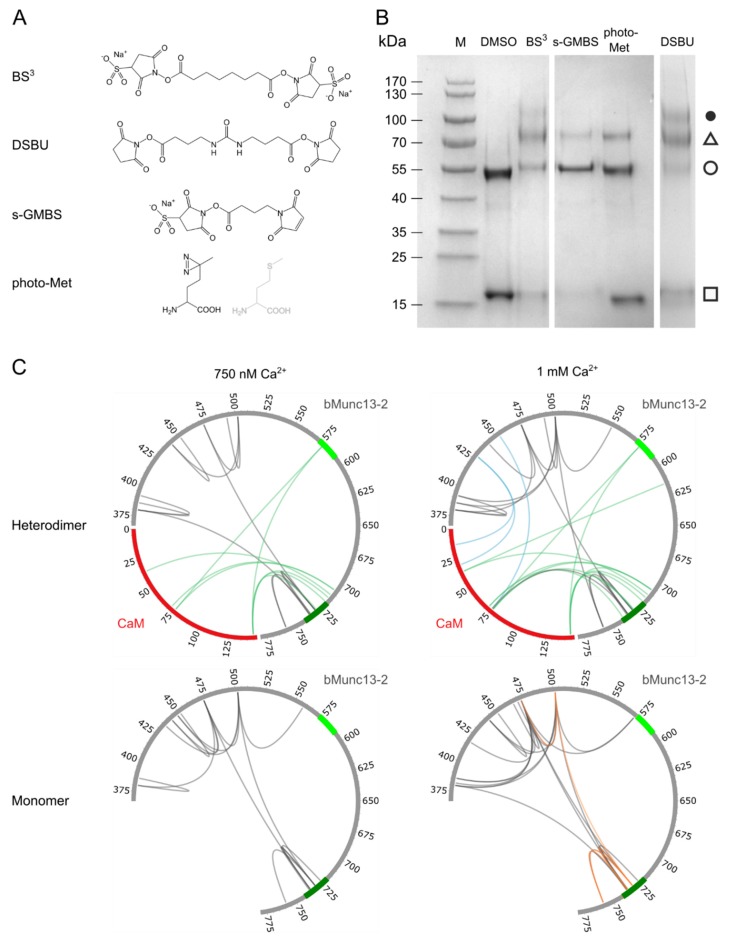
Investigation of CaM/bMunc13-2 segment-A complex by combining various cross-linking reagents. (**A**) Structures of applied cross-linking reagents (**B**) SDS-PAGE (4–20% gradient gel, Coomassie-stained) analysis of complex formation fixed by different cross-linking reagents at 1 mM Ca^2+^. (M) Prestained Protein Ladder, (□) CaM, (○) segment-A, (∆) CaM/segment-A 1:1 complex, (●) potential CaM/segment-A 2:1 complex. (**C**) Circos plots showing the identified cross-links with photo-Met (green), s-GMBS (blue), DSBU (dark gray), and BS^3^ (orange). CaM is illustrated in red and segment-A in gray. For cross-links identified with photo-Met at neighboring Met positions, the most probable one is shown. The established (aa 719–742) and predicted (aa 572–594) CaM-binding sites are shown in dark and light green, respectively (see Figure 1).

After confirming a 1:1 complex between CaM and segment-A, we performed additional cross-linking experiments to obtain more detailed insights into the three-dimensional structure of the CaM/segment-A complex. For this purpose, we applied XL-MS and used a variety of cross-linkers with different reactivities and spacer lengths (Figure 3A) to obtain complementary distance information. In addition to photo-Met, two homobifunctional, amine-reactive cross-linkers, bis(succinimidyl)suberate (BS^3^) and disuccinimidyldibutyl urea (DSBU), as well as the heterobifunctional, amine-/thiol-reactive cross-linker *N*-γ-maleimidobutyryloxysulfosuccinimide ester (s-GMBS) were applied. BS^3^ and DSBU contain two *N*-hydroxysuccinimide (NHS) esters that mainly mediate the formation of a covalent bond between primary amines in lysines within a C_α_-distance of ~30 Å [54] (Figure S3B). S-GMBS connects sulfhydryl groups in cysteines with primary amines in lysines (Figure S3C).

An overview of complex formation between CaM and segment-A with all cross-linkers used in this study is shown in Figure 3B. While the application of all reagents revealed 1:1 complexes at ~70 kDa, DSBU and BS^3^ led to the formation of an additional complex at an apparent molecular weight of ~110 kDa. As a segment-A dimer appears at an apparent molecular weight of ~130 kDa in SDS-PAGE analysis (Figure S6), the ~110 kDa band most likely represents a CaM/segment-A (2:1) complex. These 2:1 complexes only occurred with homobifunctional amine-reactive reagents of long spacer lengths, which predominantly connect Lys side chains. They were neither observed by cross-linking via photo-Met or the heterobifunctional short-spacer reagent s-GMBS, nor supported by native ESI-MS or gel electrophoresis analyses. These findings underscored the importance of applying various cross-linking reagents with different reactivity and spacer length, and led us to focus our further investigations on 1:1 complexes.

The intense CaM signal observed after cross-linking with photo-Met-CaM was due to the fact that the applied CaM is a mixture of CaM species containing none to nine photo-Met residues (variability due to incorporation of photo-Met during expression of CaM in *E. coli* [43]). Thus, the CaM species without photo-Met did not form a covalent complex with bMunc13-2 through cross-linking and were visible as single proteins in the SDS-PAGE analysis.

To identify the amino acids connected by the different cross-linkers, all SDS-PAGE signals containing the CaM/segment-A (1:1) complex (Figure 3B, triangle) were subjected to in-gel digestion. In addition, in-solution digestion was performed for the photo-Met labeled CaM/segment-A cross-linking reaction mixture. At 1 mM Ca^2+^, 16 unique intermolecular cross-linking sites between CaM and segment-A were identified with s-GMBS, DSBU, and photo-Met. Half of the cross-links were also detectable at 750 nM Ca^2+^, which is closer to the physiological concentrations (Figure 3C, upper panel; Table 1). Notably, 10 of 16 cross-links are residing within or flanking the established CaM binding site (aa 719–742) in bMunc13-2.

The highest number of cross-links was obtained with photo-Met-labeled CaM, namely 12 cross-links observed at 1 mM Ca^2+^ and eight at 750 nM Ca^2+^ (Table 1), covering six of nine possible photo-Met positions within CaM for both Ca^2+^ concentrations. When cross-linking was performed at varying Ca^2+^ concentrations (2.5 µM, 45 µM, and 300 µM), identical cross-links were observed between CaM and segment-A (Table S1). The two most prevalent cross-links observed in all samples were photo-Met76 in the central α-helix of CaM connected with E707 in bMunc13-2 and photo-Met144/145 in the *C*-terminal lobe of CaM connected with C719 in bMunc13-2 (Table S2, Figure S7).

Analysis of SDS gel bands representing the 1:1 CaM/segment-A complex and monomeric segment-A resulted in the identification of several intramolecular cross-links within segment A (Figure 3C, lower panel; Tables S3–S6). A comparison of these intramolecular cross-links indicated that segment-A adopts similar conformations in the monomeric and in the CaM-bound state at 750 nM and 1 mM Ca^2+^. Furthermore, analysis of the monomeric segment-A species at 75 nM Ca^2+^ identified intramolecular cross-links with DSBU and BS^3^ similar to those observed at higher Ca^2+^ concentrations (Table S3, Figure S8). For illustration, two exemplary spectra of DSBU cross-links are shown in Figure S9. Taken together, our cross-linking data suggest that no major Ca^2+^-induced structural changes occur in segment-A as both inter- and intramolecular cross-links were found to be highly similar at all Ca^2+^ concentrations studied.

### 3.3. Inclusion of the C1 Domain of bMunc13-2 Does Not Influence CaM Binding

To elucidate whether the presence of the C1 domain of bMunc13-2 (aa 853–903) influences bMunc13-2′s binding behavior towards CaM, we expressed a *C*-terminally elongated version of segment-A, which we termed segment-B (aa 367–903; Figure 1 and Figure S2B). We reasoned that the C1 domain might adopt a defined conformation, stabilizing the structure of segment-B. Cross-linking experiments were conducted with photo-Met-labeled-CaM, confirming the formation of a 1:1 complex in SDS-PAGE analysis (data not shown). In CaM/segment-B complexes, six intermolecular cross-links were identified that were identical to those observed for segment-A in previous experiments (Table 2, Tables S7 and S8) and also point to an interaction of CaM in vicinity to the established binding site of bMunc13-2. This similar cross-link pattern between CaM and the segment-A-specific region (aa 367–780), together with the lack of additional cross-links in the segment-B-specific region (aa 781–903), indicates that the C1 domain does not influence the structure of the bMunc13-2/CaM complex, at least not to an extent that can be resolved with this method.

### 3.4. The CaM Binding Site in bMunc13-2 Is N-Terminally Elongated

**Figure 4:**
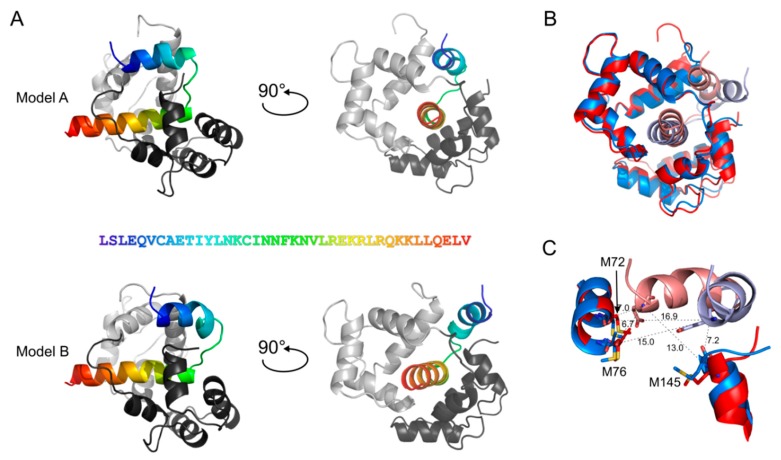
Models of bMunc13-2 segment-C (aa 704–742) with CaM generated by ROSETTA. Segment-C was docked into CaM (PDB-Code: 2O60). The *N*-terminus of bMunc13-2 segment-C interacts with the central α-helix (Model A) or the *C*-terminal amino acids of CaM (Model B). (**A**) The *N*-terminal EF-hands of CaM are colored in light gray, the *C*-terminal EF-hands are colored in dark gray; aa 704–742 of bMunc13-2 are highlighted in rainbow colors starting with blue from the *N*-terminus; the amino acid sequence is given. (**B**) Superimposition of Model A and B (RMSD of 3.04 Å). Red: Model A, blue: Model B; bright colors: segment-C, dark colors: CaM. (**C**) Zoom into the slight flexibility of the *N*-terminus in segment-C; aa 704–718 are displayed for segment-C, aa 67–77 and aa 139–148 for CaM. C_α_–C_α_ distances are shown for the cross-links connecting E707 of segment-C with the respective Met residues of CaM, which are replaced by photo-Met for cross-linking. Red: Model A, blue: Model B; bright colors: segment-C, dark colors: CaM.

Our cross-linking data with segment-A and -B not only confirmed the canonical CaM-binding site of bMunc13-2 (aa 719–742, [28,55]), but also clearly pointed to an involvement of a sequence stretch directly *N*-terminal to that site (see Circos plots in Figure 3C). When we tried to gain further structural insights by molecular modeling, we found that segment-A was not appropriate for docking studies due to its large size and absence of structurally defined domains (data not shown). Therefore, we used our cross-linking data obtained with segment-A for generating models of the complex formed between CaM and segment-C (aa 704–742) (see Figure 1 and Table 3, and details below). Modeling with the ROSETTA software [56] revealed two similar conformations for the CaM/segment-C complex, demonstrating a slight flexibility in the *N*-terminus of segment-C (Figure 4), which interacts either with aa 71–76 in the central α-helix (model A) or the *C*-terminus (model B) of CaM. This flexibility is reflected by the cross-links observed between E707 of segment-C and x71/x72, x76, or x144/x145 of CaM (Figure 4C). While still inconclusive, these models add to what is known about the structure of CaM/Munc13 peptide complexes so far [16]. According to our models, CaM remains in the compact conformation known for the minimal CaM-binding peptides of all Munc13 isoforms [28] and does not adopt the extended conformation seen with *C*-terminally elongated CaM-binding sites in the highly homologous isoforms Munc13-1 and ubMunc13-2 [32].

### 3.5. Detailed Analysis of the N-Terminally Elongated Binding Site

**Figure 5:**
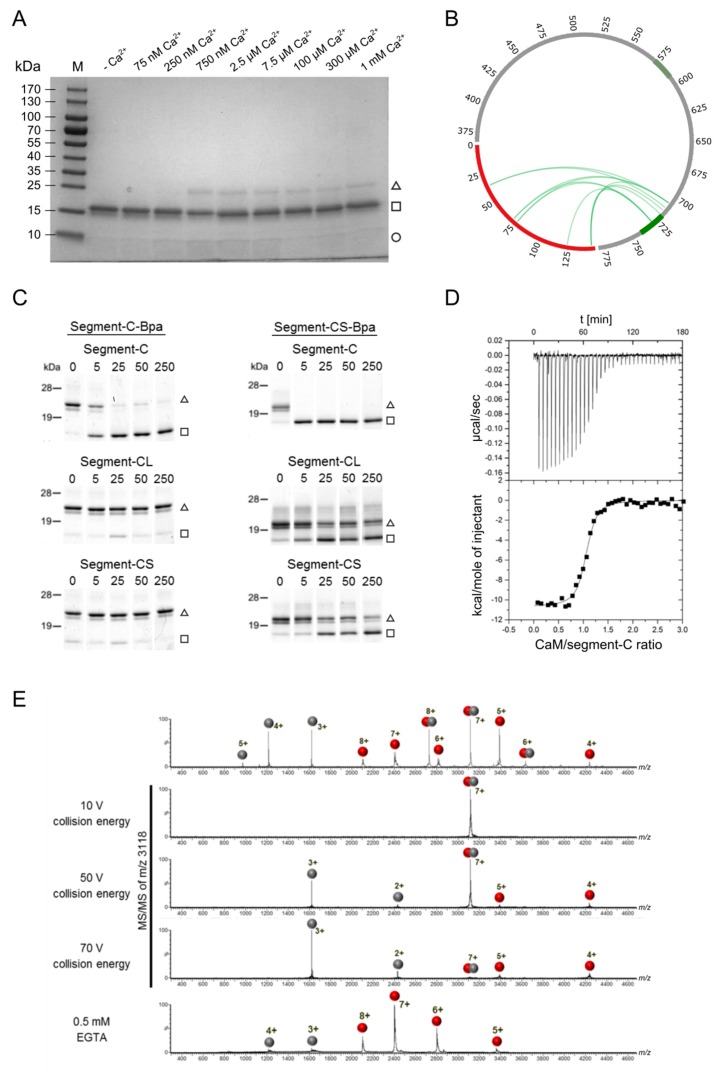
Complex formation between CaM and the segment-C. (**A**) Ca^2+^ concentration required for complex formation. The Coomassie stained gradient (4–20%) SDS-PAGE gel illustrates cross-linking of the CaM/segment-C complex at increasing Ca^2+^ concentrations. Buffer and cross-linking conditions were identical to Figure 2. The complex was covalently connected by photo-cross-linking via the incorporated photo-Met in CaM. (M) Prestained Protein Ladder, (□) CaM, (○) segment-C and (∆) CaM/segment-C 1:1 complex. (**B**) Identified cross-links between CaM and segment-C. All cross-links are illustrated as a Circos plot within the whole segment-A for comparison with previous results. CaM is colored in red, segment-A in gray and the binding sites in light and dark green within the segment-A. (**C**) PAL-based competition assay of segment-C-Bpa and segment-CS-Bpa with segment-C, -CS and -CL. (□) CaM, (∆) CaM/segment-C-Bpa or CaM/segment-CS-Bpa 1:1 complex. Complete SDS gels and corresponding mass spectra are shown in Figure S10. (**D**) Affinity measurements. ITC experiments were performed at 6.14 µM Ca^2+^, applying CaM in the reaction cell and segment-C in the syringe. A binding event was detected for N = 1.04, K_D_ = 43 ± 8 nM, ΔH = 10.68 ± 0.17 kcal·mol^−1^ and ΔS = −2.12 cal·mol^−1^·K^−1^. (**E**) Native ESI-MS. The ITC sample (CaM and segment-C) was subjected to buffer exchange prior to mass spectrometric analysis. The sample was analyzed in the absence (upper panel) and presence of EGTA (lower panel). MS/MS experiments were performed for the 7+ charged precursor ion of the complex at *m*/*z* 3118. CaM is depicted as red spheres and segment-C as gray spheres.

To further corroborate the findings from the modeling approach with experimental data, we performed XL-MS with a synthetic segment-C peptide (aa 703–742, Table 3, Figure S2C). This segment is only 15 amino acids longer than the 24 aa-minimal CaM-binding peptide used previously. Intriguingly, cross-linking experiments with segment-C revealed that the binding behavior to CaM, as well as the Ca^2+^ concentrations required for complex formation, were essentially comparable to those found for the substantially larger protein constructs segment-A and segment-B (Figure 5A, compare to Figure 2). The observation that the Ca^2+^ sensitivity for CaM binding was identical to that of segment-A was particularly unexpected because of the peptidic nature of segment-C. In accordance with the results from segment-A, the majority of the cross-links between segment-C and photo-Met-CaM (Figure 5B; Table 2, Tables S9 and S10) conclusively pointed to a region *N*-terminal to the known CaM binding motif as the major contact site. Based on these findings, we considered segment-C as a proxy for the larger protein constructs used so far. When its applicability is justified, as we feel is the case here, such a peptidic proxy increases the methodical range of options for the investigation of protein complexes and we thus relied on segment-C to further characterize the novel CaM-binding site in bMunc13-2.

We investigated the binding of segment-C to CaM with complementary approaches for macromolecular interaction analyses. We first applied a photoaffinity labeling (PAL)-based competition assay [28,55] to compare the CaM–segment-C interaction with our previous results on the minimal CaM-binding bMunc13-2 peptide (aa 719–742, termed Segment-CS hereafter). For this purpose, we generated the photoreactive variants segment-C-Bpa and segment-CS-Bpa (Table 3), in which *p*-benzoylphenylalanine (Bpa) replaces the phenylalanine at position 723 to enable photo-cross-linking reactions with CaM (Figure S3D) [28,57]. Moreover, we applied segment-CL (aa 719–752) as competitor to mirror the *C*-terminal elongation of the canonical CaM binding site, which was found in the highly homologous isoforms Munc13-1 and ubMunc13-2 [58]. When segment-C-Bpa was used as photoprobe and unmodified segment-C as competitor in increasing concentrations, suppression of photoadduct formation was found to start at equimolar concentration and to be virtually complete at a 5-fold molar excess (Figure 5C, upper left panel). In contrast, segment-CS and segment-CL could not suppress photoadduct formation (Figure 5C, middle and lower left panels), indicating that the *N*-terminal elongation of the CaM-binding site confers a higher affinity to segment-C. Inversely, photoadduct formation of the segment-CS-Bpa photoprobe was completely suppressed already in the presence of equimolar concentrations of segment-C (Figure 5C, upper right panel), indicating that segment-C basically uses the same binding site on CaM as the minimal binding peptide.

We next applied isothermal titration calorimetry (ITC) to quantify the affinity of segment-C to CaM. These measurements revealed a high affinity with a K_D_ value in the lower nanomolar range (43 ± 8 nM, Figure 5D). Native ESI-MS experiments confirmed this strong interaction as high collision energies of >50 V were required to dissociate the complex (Figure 5E). The Ca^2+^ dependence of the interaction between CaM and segment-C was confirmed by dissociating the complex by adding EGTA (Figure 5E, lower panel).

Taken together, our interaction analyses showed that segment-C binds CaM with an affinity in the nanomolar range and that this high affinity is conferred to bMunc13-2 by the sequence stretch aa 703–718 located *N*-terminally to the canonical CaM binding site.

### 3.6. Hydrophobic Contact Sites in the N-Terminal Part of the bMunc13-2 CaM Binding Sequence Are Crucial for High-Affinity Binding

To further characterize the involvement of the *N*-terminal part of segment-C in high-affinity CaM binding, specific mutations were introduced at positions V709, I714 and F723 where potential hydrophobic anchor residues were exchanged to aspartic acid (Table 3). Aspartic acid residues are frequently introduced into CaM target proteins to study their interactions at the molecular level by replacing hydrophobic or basic amino acids in putative CaM binding motifs [59,60,61,62].

**Figure 6:**
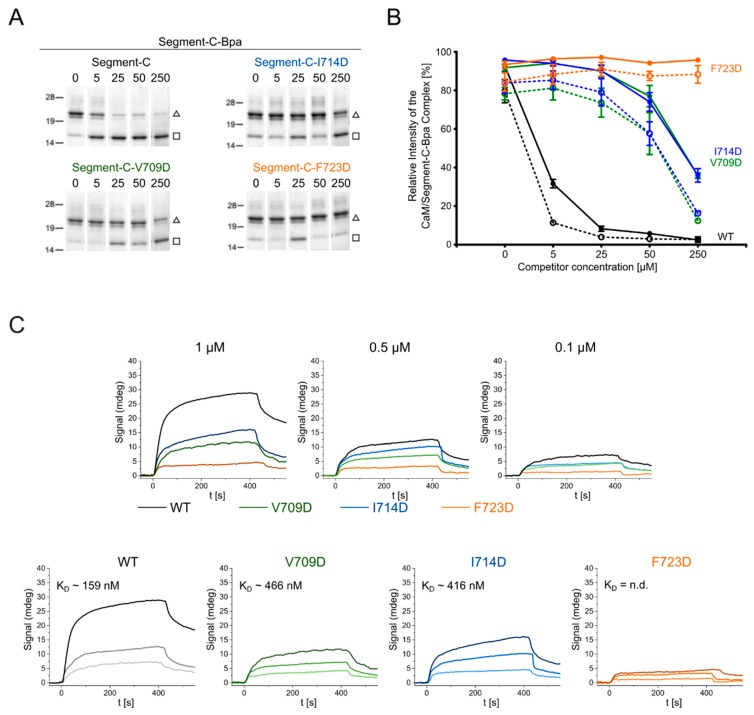
CaM/segment-C interaction studies by photoaffinity labeling and surface plasmon resonance. Amino acid sequences of segment-C variants are given in Table 3. (**A**,**B**) PAL-based competition assay of segment-C-Bpa with segment-C and its variants V709D (green), I714D (blue), and F723D (orange). (**A**) SDS-PAGE analysis of photoreactions; (□) CaM, (∆) CaM/segment-C-Bpa 1:1 complex. (**B**) quantification of photoadduct yield (CaM/segment-C-Bpa signal); solid lines, quantification by SDS-PAGE analysis; dotted lines, quantification by MALDI-TOF-MS. The plot shows the mean of three independent experiments with standard error of mean (SEM) as error bars. A representative set of SDS gels and corresponding mass spectra is shown in Figure S11. (**C**) SPR measurements of wild-type (WT) and mutated segment-C variants V709D, I714D and F723D. Upper panel: sensograms of all variants for one concentration, lower panel: sensograms of one segment-C variant at different concentrations. K_D_ values are given; n.d.: not determined.

First, PAL competition assays were performed, in which segment-C-Bpa was used as photoprobe, while segment-C and its mutant variants, V709D, I714D, and F723D, served as competitors (Figure 6A,B). As observed before, a five-fold molar excess of segment-C was sufficient to fully displace its Bpa-labeled counterpart from CaM binding. In contrast, even 50-fold higher concentrations of the mutant variants, V709D and I714D, were not sufficient to reach the same level of displacement. Notably, the variant F723D was not able to displace segment-C-Bpa at all, even not when applied in 50-fold molar excess. Although of a qualitative nature, our data indicate that all three hydrophobic residues exchanged, V709, I714, and F723, contribute to the high CaM-binding affinity of segment-C, with the phenylalanine residue in position 723 obviously functioning as a crucial hydrophobic anchor residue.

To obtain quantitative binding data on the novel CaM binding site and to further characterize the molecular determinants underlying high-affinity binding, surface plasmon resonance (SPR) measurements were performed with segment-C and its mutant variants V709D, I714D, and F723D. For this purpose, biotinylated CaM (Figure S4) was immobilized on a chip and increasing concentrations of segment-C and its variants (0.1 µM, 0.5 µM, and 1 µM) were applied to the CaM-coated surface. The recorded sensograms are shown in Figure 6C. For segment-C, a high affinity to CaM (K_D_ = 159 nM) was determined, agreeing well with the ITC data (Figure 5D). Exchanging the hydrophobic residues V709 and I714 with aspartic acid resulted in an approximately 3-fold decrease of affinity, while a K_D_ value could not be derived for the F723D variant. Overall, our findings from the competition assays and SPR experiments converged to establish hydrophobic contacts between CaM and the *N*-terminal part of the CaM binding site as crucial determinants of its high binding affinity.

**Figure 7:**
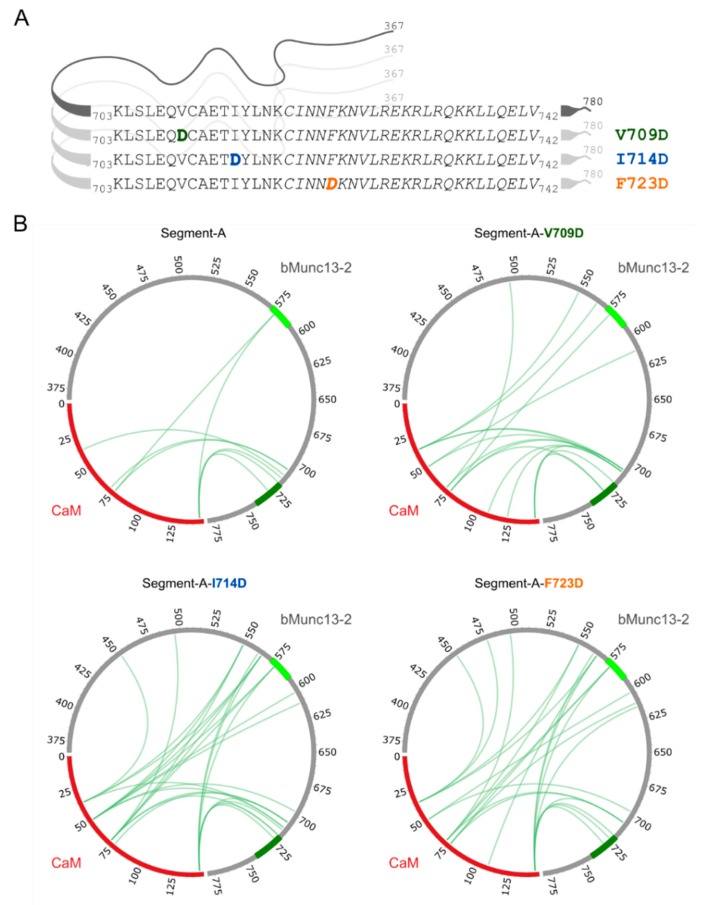
Segment-A variants and photo-Met cross-linking with CaM. (**A**) Segment-A variants (V709, I714 and F723) were generated by exchanging hydrophobic amino acids to aspartic acid (bold, colored letters). Displayed are the amino acid sequences of the previously employed segment-C, in which the proposed CaM binding region is shown in italics. (**B**) Cross-links observed with photo-Met for wild-type and segment-A variants (750 nM Ca^2+^). CaM is highlighted in red; CaM binding sites are colored in light green (predicted site, aa 572–594) and dark green (established site, aa 719–742) within segment-A (gray).

Finally, we tested the significance of amino acid residues V709, I714 and F723 for CaM binding in the protein context. For this purpose, these hydrophobic amino acids were exchanged to aspartic acid in segment-A (Figure 7A). Photo-cross-linking experiments were performed with photo-Met-labeled CaM at 750 nM Ca^2+^ applying the conditions as described above. The cross-link sites found for segment-A and its mutant variants (Figure 7B, Tables S11–S13) revealed that the cross-linked amino acids are located in the region of aa 703–742 in bMunc13-2. However, an increased number of cross-links involving other regions of segment-A were found for the mutant variants in general and for the F723D variant in particular (Figure 7B, Figure S12). These subordinate binding sites included, but were not limited to the further *N*-terminally located candidate CaM binding site in bMunc13-2 (aa 572–594), which was predicted by bioinformatics tools, but shown to be deficient in binding in the protein context [28]. Although we cannot rule out the formation of 2:1 CaM/segment-A complexes when CaM is applied in large excess, we found a predominant occurrence of 1:1 complexes irrespective of the detection method applied (cross-linking-based versus native), which led us to conclude that the predicted binding site is capable of CaM binding only if the high-affinity binding site is compromised. This is the case in the mutant variants due to the absence of hydrophobic anchor residues. Thus, the lower affinity of the variants may lead to conformational promiscuity in the bound state, which is ultimately reflected by scattered cross-linking events.

## 4. Discussion

We have previously shown that all four Munc13 isoforms are regulated by Ca^2+^/CaM binding to a CaM-binding site located at a similar position *N*-terminally of their C1 domains [28]. While the position of the CaM-binding site appears to be conserved throughout the entire protein family, the type of CaM-binding motif and the structural features of the interaction are not. The most closely related isoforms, Munc13-1 and ubMunc13-2, bind CaM via a *C*-terminally extended motif of the 1–5–8–26 type [32], but no such detailed information was available for the brain-specific isoform bMunc13-2 exhibiting the strongest CaM-dependent effects on synaptic plasticity in vitro [28].

To study the CaM-bMunc13-2 interaction, we used two different constructs from the *N*-terminal region of bMunc13-2. Both, segment-A (aa 367–780) and segment-B (aa 367–903) formed complexes with CaM in a 1:1 stoichiometry already at apparent Ca^2+^ concentrations in the high nanomolar range, rendering these proteins appropriate to structurally characterize CaM binding behavior under at least pseudo-physiologic conditions: only XL-MS experiments with segment-A revealed the *N*-terminal extension of the canonical CaM-binding motif in bMunc13-2 (aa 719–742), a unique feature of this Munc13 isoform that had been overlooked in previous experiments with short CaM-binding peptides.

**Figure 8:**
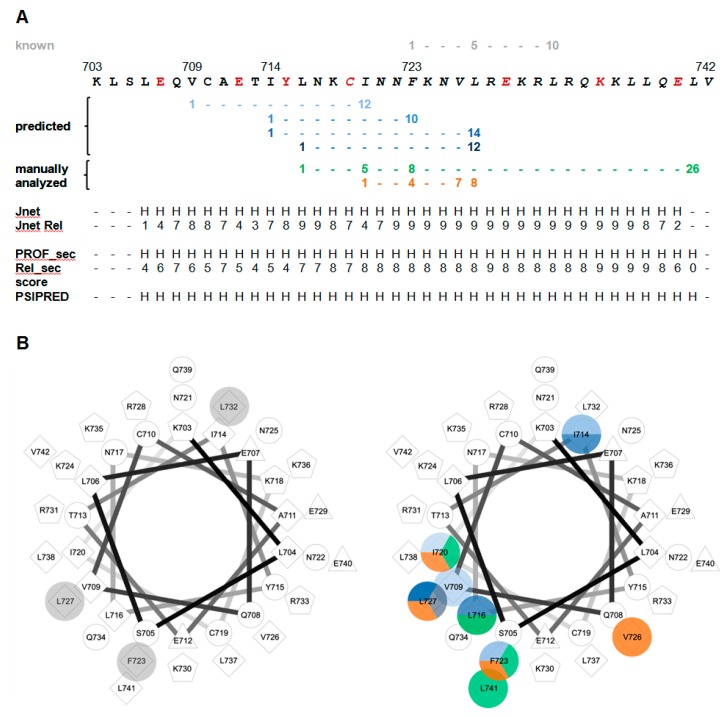
Analysis of potential binding motifs in bMunc13-2 segment-C. Only CaM binding motifs starting *N*-terminally to position 1 of the proposed 1–5–10 binding motif were analyzed. The known 1–5–10 CaM binding motif is colored in gray, CaM binding motifs identified by database comparison are shown in different shades of blue, manually identified CaM binding motifs are colored green and orange. (**A**) Amino acid sequence, predicted CaM binding motifs and secondary structure prediction of segment-C. Amino acids identified in cross-linking studies are highlighted in red. Three different prediction servers were used: Jpred (www.compbio.dundee.ac.uk/jpred), PredictProtein (www.predictprotein.org), and PSIPRED (bioinf.cs.ucl.ac.uk/psipred). The algorithms Jnet and PROF_sec provide a score for α-helical propensity between 0 (lowest propensity) and 9 (highest propensity). H, predicted to be α-helical. (**B**) Helical wheel projection of segment-C. The known CaM binding motif is displayed on the left; novel CaM binding motifs are highlighted on the right.

The constraints derived from XL-MS with segment-A served as basis for modeling studies between CaM and segment-C (aa 704–742), a peptide that extends the minimal CaM-binding peptide from bMunc13-2 by 15 aa towards the *N*-terminus and that is predicted to form a straight α-helix (Figure 8A). Interestingly, modeling resulted in two similar conformations for the complex due to the flexibility in the positioning of the *N*-terminus of segment-C. This flexibility is most likely enabled by a kink in the α-helix of segment-C, which divides segment-C into an *N*-terminal (~10–15 aa) and a *C*-terminal α-helical part. Formation of such a kink in the *N*-terminal part of segment-C might be facilitated by a drop in propensity to form a α-helix in that region of the peptide, as indicated by lower prediction scores for helix formation (Figure 8A).

The flexible part of segment-C (i.e., the *N*-terminal helix) can either interact with the central α-helix or the *C*-terminus of CaM, as reflected by the cross-links observed between E707 of bMunc13-2 and x71/x72, x76, or x144/x145 of CaM. Irrespective of this flexibility, the complex between the bMunc13-2 segment-C and CaM fails to adopt an extended conformation. This contrasts previous findings for the highly homologous isoforms Munc13-1 and ubMunc13-2 where extended structures were observed for CaM-binding, leading to the definition of the 1–5–8–26 CaM-binding motif [32].

To further characterize the CaM binding site of bMunc13-2, we introduced here the peptide segment-C (aa 703–742) and showed that the Ca^2+^ sensitivity for CaM binding and the majority of the cross-links were identical to that of the much longer segment-A. Thus, we considered segment-C as a valid peptidic proxy for the investigation of the CaM binding behavior of bMunc13-2. Indeed, our interaction analyses showed that segment-C binds CaM with an affinity in the nanomolar range. Strikingly, this high affinity is conferred to bMunc13-2 by the sequence stretch comprising aa 703–718, i.e., the *N*-terminal extension of the canonical 1–5–10 CaM-binding motif. Specifically, residues V709 and I714, in addition to the putative anchor residue F723, were identified as crucial determinants of high-binding affinity by amino acid exchanges introduced in segment-C and segment-A. As secondary structure predictions indicated that these amino acid exchanges did not change the overall helicity (data not shown), we conclude that the residues exchanged are indeed hydrophobic contact sites mediating the interaction of segment-C with CaM.

A comparison of the newly identified hydrophobic interaction sites with the canonical 1–5–10 CaM-binding motif (and its closely related variants 1–10, 1–12, and 1–14 identified by a database search), with the 1–5–8–26 motif known from Munc13-1/ubMunc13-2, and with the 1–4–7–8 motif recently discovered in AKAP79 [63], indicates that none of the known or predicted binding motifs can account for the newly identified *N*-terminal contact sites (Figure 8). Additionally, the 1–5–8–26 and 1–4–7–8 CaM-binding motifs differ in their hydrophobic anchor amino acids. In Munc13-1, ubMunc13-2 and AKAP79, the first position of the CaM-binding motif is represented by the bulky, hydrophobic amino acid Trp, while in bMunc13-2, the first position is a Leu or Ile residue. The same applies for position 26 within the 1–5–8–26 motif, which in addition is localized differently in comparison to the aligned CaM binding site region of Munc13-1 and ubMunc13-2 (Figure S13, [32]).

Visualization of the candidate CaM-binding motifs in a helical wheel projection indicates that the potential hydrophobic anchor residues of the 1–5–8–26 and the 1–4–7–8 motifs fall together into neighboring regions at the same side of the α-helix, which could indicate an extended conformation of CaM while binding to bMunc13-2 (Figure 8). In support of our models created for the segment-C/CaM complex, an α-helix with a kink can be conceived for bMunc13-2, potentially fulfilling the molecular requirement for the extended conformation typical for CaM complexes of the 1–5–8–26 type [32]. However, we did not find any evidence by XL-MS for an extended conformation. Taken together, we consider the 1–5–8–26 motif as unlikely candidate for the CaM-binding motif of bMunc13-2. We then extended the analysis of potential CaM-binding motifs to all Munc13 isoforms and complemented it by secondary structure predictions (Figure S13). Overall, we realized that several candidate CaM binding motifs seem to be compatible with the respective region of bMunc13-2, probably related to its high content of hydrophobic amino acids. Most interesting, however, was the observation of a predicted helical structure starting more *N*-terminally compared to the other Munc13 isoforms. This helix, although predicted to be straight, likely includes a kink as indicated by our models and further supported by a local drop in the predicted propensity for helix formation (Figure 8A). These observations strengthen the existence of a unique *N*-terminally extended CaM binding motif in bMunc13-2. We thus propose the presence of a novel CaM-binding motif with additional hydrophobic contact sites *N*-terminally of aa 719–742 of bMunc13-2. So far, the positions indicate a 1–6–15 motif, which has to be validated in further studies including high-resolution structural analysis. The fact that the proposed hydrophobic positions (V709, I714, and F723) as well as the secondary structure prediction are conserved from zebrafish to rats to humans and differ from the other isoforms Munc13-1, ubMunc13-2 and Munc13-3 (Figures S14 and S15) further substantiates our finding of a novel CaM binding motif in bMunc13-2.

## 5. Conclusions

We demonstrate that the interaction between CaM and bMunc13-2 is unique among the Munc13 isoforms and mediated by a novel CaM binding site, which is an *N*-terminal extension of the known CaM 1-5-10 binding motif. Munc13-1 and ubMunc13-2 exhibit a 1–5–8–26 motif [32,64], which is a *C*-terminal extension of a classical, 1–5–8 CaM binding motif. It remains to be studied whether dimension and type of the CaM-dependent effect on the function of different Munc13 isoforms are related to the mode of CaM interaction via an *N*- or a *C*-terminally extended CaM binding motif.

## Figures and Tables

**Table 1:** Intermolecular cross-links identified between CaM and the bMunc13-2 segment-A at 750 nM and 1 mM Ca^2+^. x denotes photo-Met; Y, cross-link detected; N, cross-link not detected.

Cross-Linker	Cross-Linked Amino Acid CaM	Cross-Linked Amino Acid Segment-A	Ca^2+^ Concentration	
			750 nM	1 mM
**Photo-Met**	x36	D615	N	Y
	x36	E712	Y	Y
	x71/x72	E707	Y	Y
	x71/x72	E740	N	Y
	x76	H578	Y	Y
	x76	E707	Y	Y
	x76	E729	N	Y
	x144/x145	H578/H579	Y	Y
	x144/x145	E707	N	Y
	x144/x145	Y715	Y	Y
	x144/x145	C719	Y	Y
	x144/x145	E729	Y	Y
**DSBU**	K75	K735	N	Y
**s-GMBS**	K75	C453	N	Y
	K13	C428	N	Y
	K30	C428	N	Y

**Table 2:** Comparison of intermolecular cross-links identified between CaM and bMunc13-2 segments. The constraints derived from the cross-links with segment-A and segment-B were used for structural modeling. x denotes photo-Met.

Cross-Linked Amino Acid CaM	Cross-Linked Amino Acid bMunc13-2	Segment-A (aa 367–780)	Segment-B (aa 367–903)	Segment-C (aa 703–742)
x36	E707			✓
x36	D615	✓		
x36	E712	✓		✓
x71/x72	E707	✓	✓	✓
x71/x72	E729			✓
x71/x72	E740	✓	✓	
x76	H578	✓		
x76	E707	✓	✓	✓
x76	E729	✓		✓
x124	E729			✓
x144/x145	H578/H579	✓		
x144/x145	E707	✓	✓	✓
x144/x145	Y715	✓	✓	✓
x144/x145	C719	✓	✓	✓
x144/x145	E729	✓		✓

**Table 3:** Amino acid sequences of segment-C variants. Summary of segment-C variants employed in the experiments shown in Figure 5 and Figure 6. The replacement by aspartate is colour-coded in the respective variants: green for V709D, blue for I714D, orange for F723D. The canonical CaM binding region [28] is shown in italics; f denotes Bpa.

Amino Acid Sequences of Segment-C	Variants of Segment-C
_703_KLSLEQVCAETIYLNK*CINNFKNVLREKRLRQKKLLQELV*_742_	Segment-C
_703_KLSLEQVCAETIYLNK*CINNfKNVLREKRLRQKKLLQELV*_742_	Segment-C-Bpa
_719_ *CINNFKNVLREKRLRQKKLLQELV*_742_	Segment-CS
_719_ *CINNfKNVLREKRLRQKKLLQELV*_742_	Segment-CS-Bpa
_719_*CINNFKNVLREKRLRQKKLLQELV*QTASHLSVED_742_	Segment-CL
_703_KLSLDQVCAETIYLNK*CINNFKNVLREKRLRQKKLLQELV*_742_	Segment-C-V709D
_703_KLSLEQVCAETDYLNK*CINNFKNVLREKRLRQKKLLQELV*_742_	Segment-C-I714D
_703_KLSLEQVCAETIYLNK*CINNDKNVLREKRLRQKKLLQELV*_742_	Segment-C-F723D

## Supplementary Materials

The following are available online at https://www.mdpi.com/2073-4409/9/1/136/s1, [65,66]: I, Supplementary Figures S1–S15; II, Supplementary Tables S1–S13; III, Supplementary Methods—Protocol capture page for modeling approach.
