# Supplementary material for: Delineating the Molecular Basis of the Calmodulin–bMunc13-2 Interaction by Cross-Linking/Mass Spectrometry—Evidence for a Novel CaM Binding Motif in bMunc13-2
Source: Cells. 2020 Jan 7;9(1):136. doi: 10.3390/cells9010136 (PMC7017353; doi:10.3390/cells9010136)
Supplement: Supplementary file 1 [file cells-09-00136-s001.zip › Cells-661696_Supplementary_files/Cells-661696_I-SupplFigs.pdf]

SUPPLEMENTARY MATERIAL I

Supplementary Figures

Delineating the Molecular Basis of the Calmodulin/bMunc13-2 Interaction by Cross-linking/Mass Spectrometry – Evidence for a Novel CaM Binding Motif in bMunc13-2

Christine Piotrowski[1], Rocco Moretti[2], Christian H. Ihling[1], André Haedicke[3,†], Thomas Liepold[4], Noa Lipstein[5], Jens Meiler[2], Olaf Jahn[4,*], Andrea Sinz[1,*]

[1] Department of Pharmaceutical Chemistry and Bioanalytics, Institute of Pharmacy, Charles Tanford Protein Center, Martin Luther University Halle-Wittenberg, D-06120 Halle/Saale, Germany;

[2] Center for Structural Biology, Department of Chemistry, Vanderbilt University, Nashville, TN 37221, USA

[3] Biophysical Chemistry, Institute of Chemistry, Martin Luther University Halle-Wittenberg, D-06120 Halle/Saale, Germany

[4] Proteomics Group, Max Planck Institute of Experimental Medicine, D-37075 Göttingen, Germany

[5] Department of Molecular Neurobiology, Max Planck Institute of Experimental Medicine, D-37075 Göttingen, Germany

* Correspondence: jahn@em.mpg.de (O.J.); andrea.sinz@pharmazie.uni-halle.de (A.S.); Tel.: +49-551-3899-313 (O.J.); +49-345-5525170 (A.S.)

† Present address: Serumwerk Bernburg AG, D-06406 Bernburg, Germany.

```
001 ADQLTEEQIA EFKEAFSLFD KDGDGTITTK ELGTVMRSLG QNPTEAELQD MINEVDADGN
061 GTIDFPEFLT MMARKMKDTD SEEEIREAFR VFDKDGNGYI SAAELRHVMT NLGEKLTDEE
121 VDEMIREADI DGDGQVNYEE FVQMMTAKLE HHHHHH

    MW = 17,771 Da
```

Figure S1: Amino acid sequence of recombinant, C-terminally $(His)_6$-tagged CaM. Met residues can be replaced by photo-Met (1).

A

GSPEFHQGL SFLPKDGSAK QSDVSKLQDE VKGTSGAPQV ISDPCGELSL LHQLEGSSPV
421 LIPKEEDCGK LQIFKQDSQE HKACNVTKLQ SDCNNAIKAS SCLSLSGPLK AEKVNAEDRM
481 LGGEDGLDIL SPKQLEDLLA DKSRRFATLN PDSAVEEVII GPETFSNMVH IDLNEEETCT
541 AQVLKNVFDK SSCVLGGSQE DEDVEIKFHT TKLSRAIHHF RLALQGVFQK LENNGSISPE
601 DLESNESGSQ SENSDRLLWT VSSGGAHDCS VESPASQGSE SLLSVVSGGV GISVQGDQTP
661 QAPSNFSLAS NNSPLTNSLL SFPLAPGLGN ETCSRPDSPN QGKLSLEQVC AETIYLNK**CI**
721 **NNFKNVLREK RLRQKKLLQE LV**QTASHLSV EDIPSEGKRE ALQISDDGDP SLPQWLPEGP
LERPHRD

MW = 46,032 Da

B

GSHQGL SFLPKDGSAK QSDVSKLQDE VKGTSGAPQV ISDPCGELSL LHQLEGSSPV
421 LIPKEEDCGK LQIFKQDSQE HKACNVTKLQ SDCNNAIKAS SCLSLSGPLK AEKVNAEDRM
481 LGGEDGLDIL SPKQLEDLLA DKSRRFATLN PDSAVEEVII GPETFSNMVH IDLNEEETCT
541 AQVLKNVFDK SSCVLGGSQE DEDVEIKFHT TKLSRAIHHF RLALQGVFQK LENNGSISPE
601 DLESNESGSQ SENSDRLLWT VSSGGAHDCS VESPASQGSE SLLSVVSGGV GISVQGDQTP
661 QAPSNFSLAS NNSPLTNSLL SFPLAPGLGN ETCSRPDSPN QGKLSLEQVC AETIYLNK**CI**
721 **NNFKNVLREK RLRQKKLLQE LV**QTASHLSV EDIPSEGKRE ALQISDDGDP SLPQWLPEGP
781 AGGLYGIDSM PDLRRKKPLP LVSDLAMSLV QSRKAGITSA MATRTSLKDE DLKSHVYKKT
841 LQALIYPISC TTPHNFEVWS ATTPTYCYEC EGLLWGLARQ GMRCSECGVK CHEKCQDLLN
901 ADCEFPGRLE RPHRD

MW = 59,859 Da

C

703 *K*LSLEQVC AETIYLNK**CI** **NNFKNVLREK RLRQKKLLQE LV**

MW = 4,854 Da

Figure S2: Amino acid sequences of bMunc13-2 segments. (A) Segment-A; (B) segment-B; (C) segment-C with acetylated *N*-terminus. K703 was used for peptide synthesis, but not for modeling. Underlined: known CaM binding site (aa 572-594), **bold and underlined**: known 1-5-10 CaM binding site (aa 719-742), dashed: amino acids originating from the multiple cloning site.

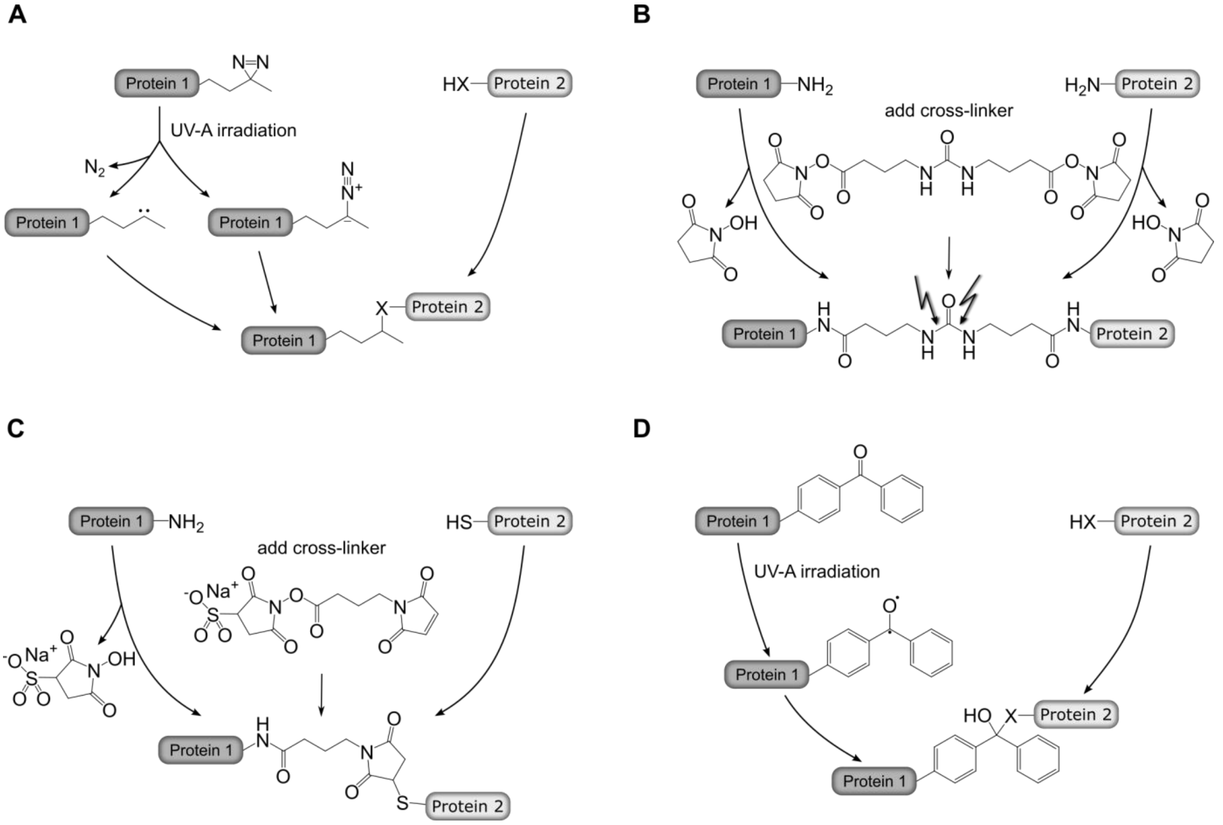

Figure S3: Reaction mechanisms of cross-linkers used in this study. (A) Photo-Met, (B) homobifunctional, amine-reactive DSBU cross-linker; arrows indicate MS-cleavability, (C) heterobifunctional, amine-/sulfhydryl-reactive cross-linker s-GMBS, (D) photo-reactive amino acid Bpa.

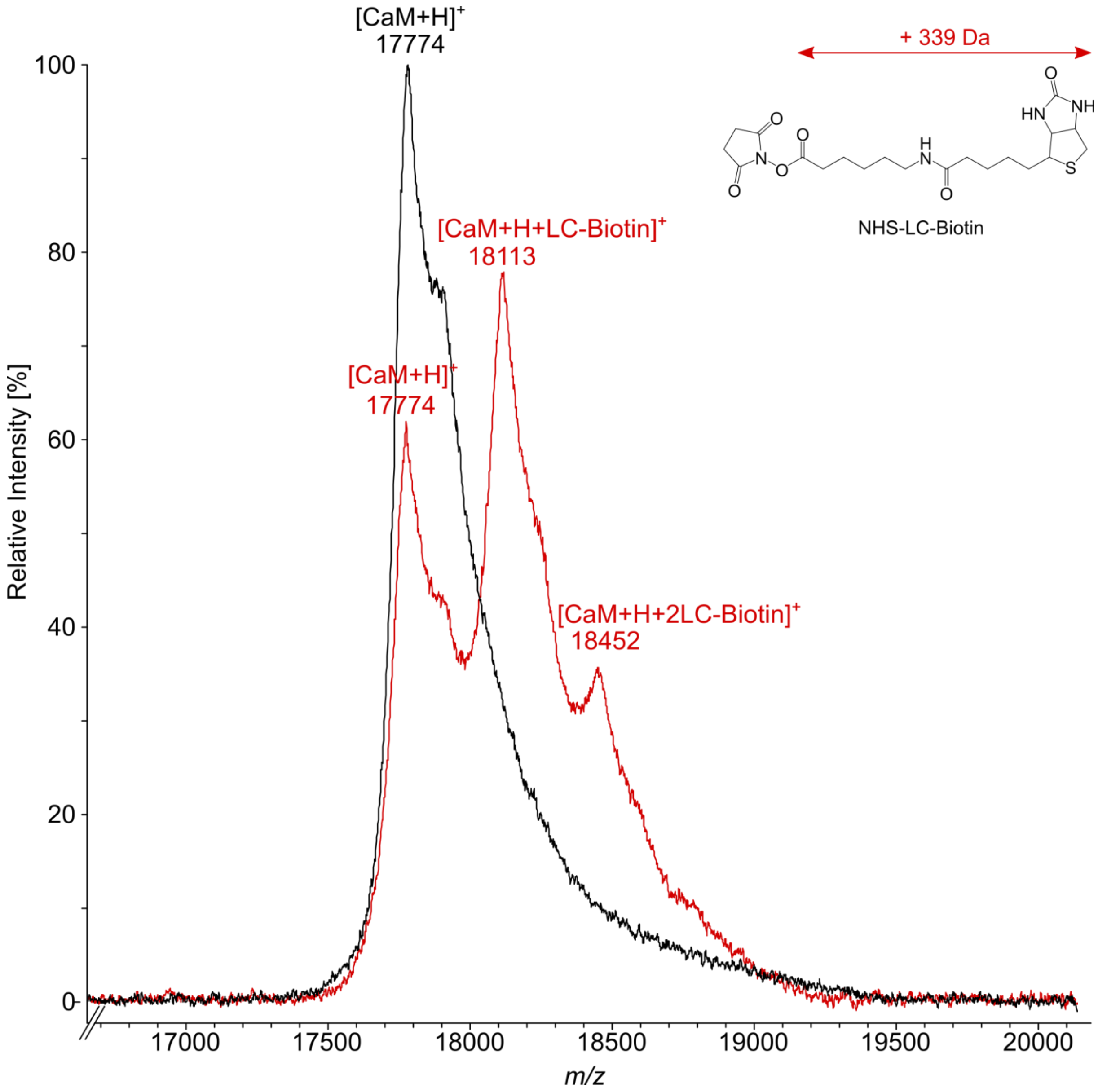

Figure S4: MALDI-TOF-MS of biotinylated photo-Met-CaM. Photo-Met CaM (black signal) and biotinylated (with LC-Biotin) photo-Met-CaM (red signal) were subjected to MALDI-TOF-MS analysis after treating the protein solutions with C4-ZipTip pipette tips (Merck Millipore, Darmstadt, Germany) according to the manufacturer's protocol. Protein solutions were co-crystallized with the MALDI matrix super-dihydroxybenzoic acid (DHB) on a steel MALDI target. The *m/z* values of the peaks are displayed. Biotinylation with LC-Biotin induces a mass shift of 339 u.

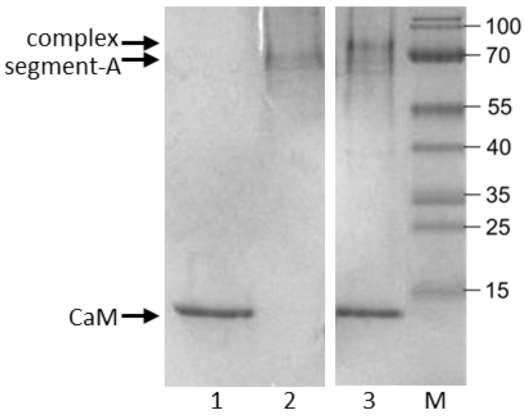

Figure S5: Native PAGE analysis of the CaM/bMunc13-2-segment-A interaction. CaM and bMunc13-2 segment-A were incubated in equimolar ratio at 1 mM Ca^{2+}. The reaction mixture was analyzed by native gel electrophoresis (lane 3) and its electrophoretic mobility was compared with that of the individual proteins alone (CaM, lane 1; segment-A, lane 2). In lane 3, a distinct signal for the segment-A/CaM complex is visible at a molecular weight best compatible with a 1:1 stoichiometry. Note that a 1:2 (segment-A:CaM) complex is not evident despite the availability of further free CaM. M: prestained molecular ruler for SDS-PAGE analysis.

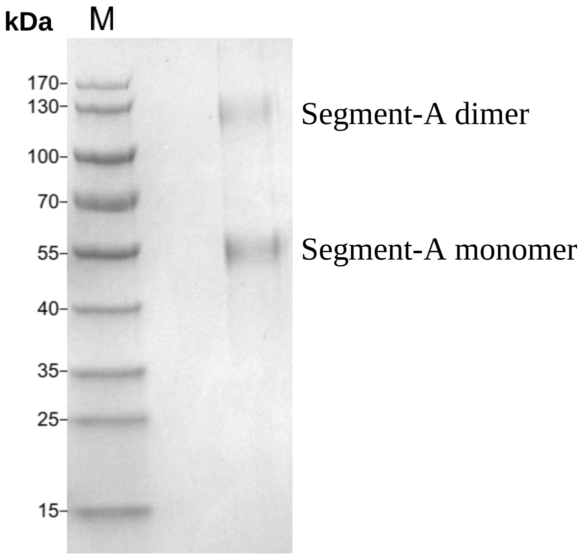

Figure S6: BS^3-cross-linking of bMunc13-2 segment-A. SDS-PAGE analysis (4-20% gradient gel, Coomassie-stained) of cross-linked segment-A. Cross-linking was performed at 25°C for 60 min with a 100-fold excess of BS^3 over protein concentration. M: Prestained Protein Ladder.

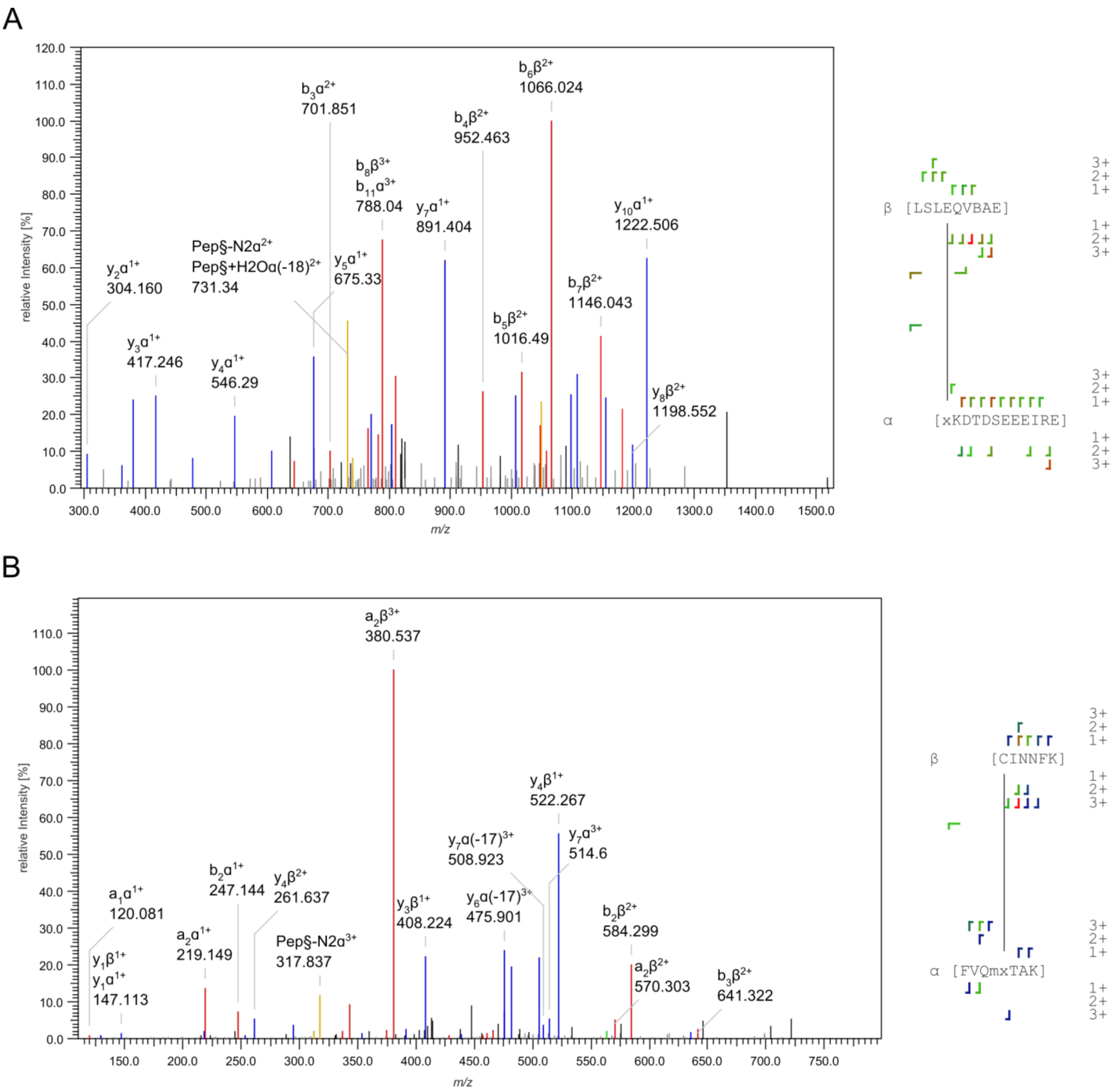

Figure S7: Fragment ion mass spectra of photo-Met cross-links between CaM and bMunc13-2 segment-A. x denotes photo-Met; m denotes oxidized Met. (A) Cross-link between x76 of CaM and E707 of segment-A comprising amino acids 76-87 of CaM and 704-712 of segment-A. Precursor: *m/z* 837.059, charge state 3+, $[M+H]^+_{theo}$ = 2509.161. (B) Cross-link between x144/x145 of CaM and C719 of segment-A comprising amino acids 141-148 of CaM and 719-724 of segment-A. Precursor: *m/z* 422.968, charge state 4+, $[M+H]^+_{theo}$ = 1688.850. The peaks are labeled as follows: red: a- or b-type ions, blue: y-type ions, green: precursor ions, yellow: ions generated by specific cross-linker cleavage, §: peptide containing photo-Met and #: peptide without photo-Met.

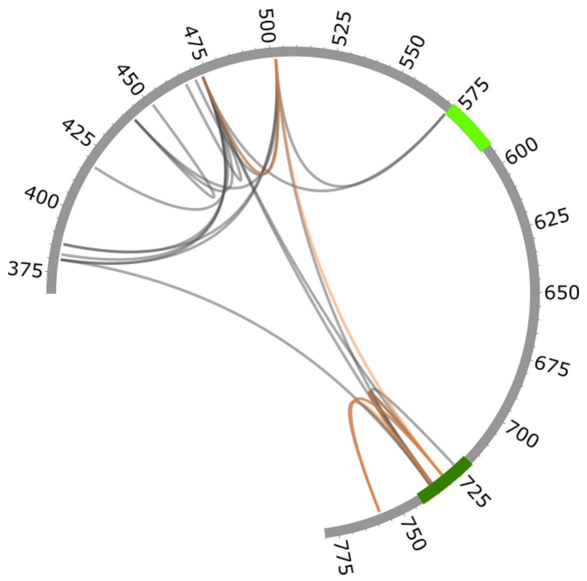

Figure S8: Intramolecular cross-links within bMunc13-2 segment-A (75 nM Ca^{2+}). The cross-links were identified from the monomer of segment-A (SDS-PAGE comparable to Figure S5). Light/dark green: CaM binding sites, orange: BS^3, grey: DSBU.

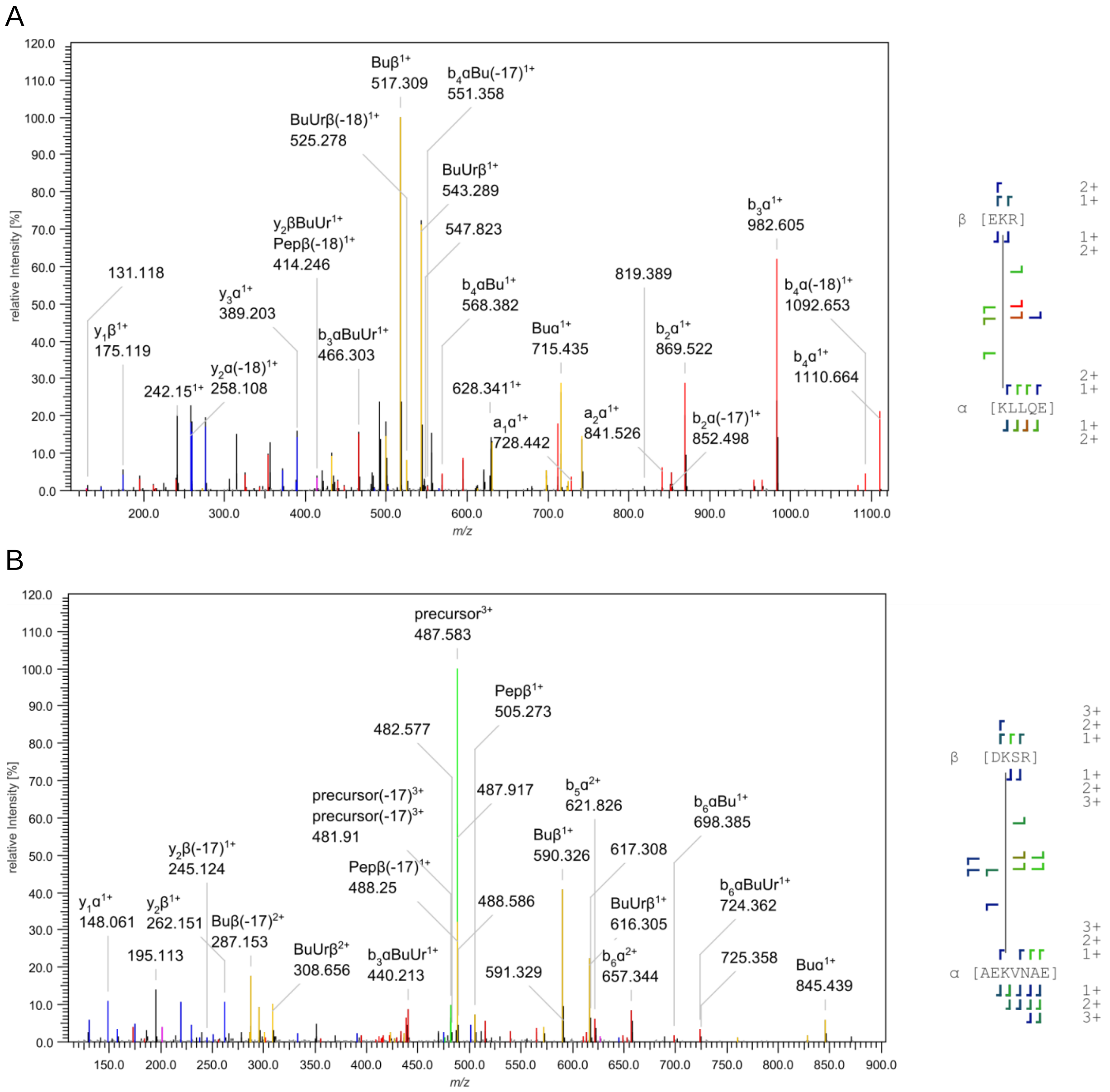

Figure S9: Fragment ion mass spectra of intramolecular DSBU-cross-links within bMunc13-2 segment-A. (A) Cross-link between K730 and K736 of segment-A comprising amino acids 729-731 and aa 736-741 of segment-A. Precursor: *m/z* 629.362, charge state 2+, $[M+H]^+_{theo}$ = 1257.716. (B) Cross-link between K473 and K502 of segment-A comprising aa 471-479 and aa 501-504 of segment-A. Precursor: *m/z* 487.583, charge state 3+, $[M+H]^+_{theo}$ = 1460.734. The peaks are labeled as follows: red: a- or b-type ions, blue: y-type ions, green: precursor ions, yellow: ions generated by specific cross-linker cleavage.

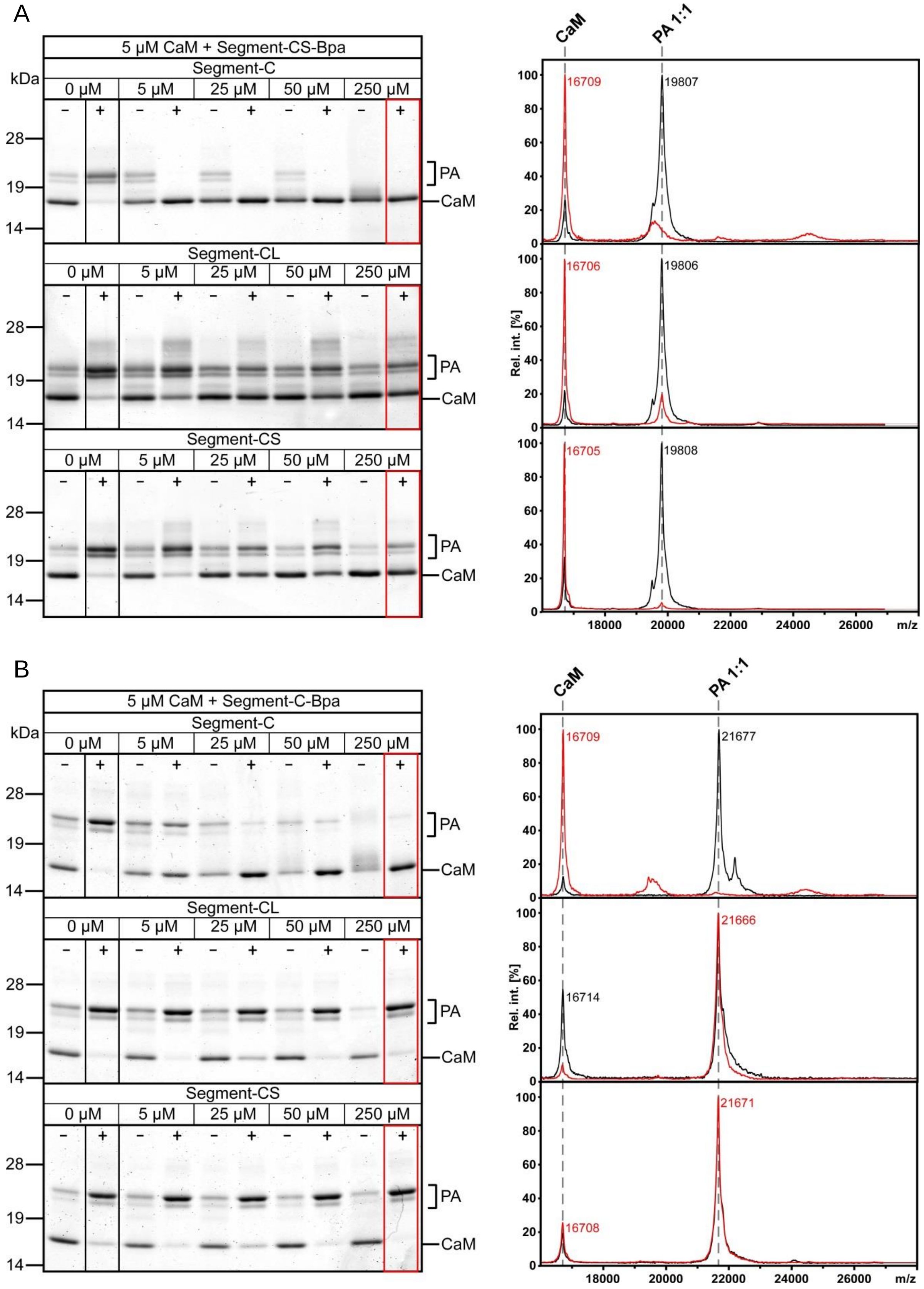
A
5 µM CaM + Segment-CS-Bpa
Segment-C
kDa
0 µM
5 µM
25 µM
50 µM
250 µM
28
19
14
PA
CaM
Segment-CL
Segment-CS
CaM
PA 1:1
16709
19807
16706
19806
16705
19808
Rel. int. [%]
18000
20000
22000
24000
26000
m/z
B
5 µM CaM + Segment-C-Bpa
16709
21677
21666
16714
21671
16708

Figure S10: SDS-PAGE and MALDI-TOF-MS analysis of PAL-based competition assays. (A) bMunc13-2 segment-C-Bpa, (B) bMunc13-2 segment-CS-Bpa, (-) sample without Ca^{2+}, (+) sample containing 200 µM Ca^{2+}. The left panels display the SDS-PAGE analysis; the right panels show the mass spectra of the formation of the CaM/bMunc13-2 complex. The black trace in the mass spectra refers to the samples boxed in black in the gel image (presence of Ca^{2+}, absence of competitor); the red trace in the mass spectra refers to the samples boxed in red in the gel image (presence of Ca^{2+}, presence of competitor in 50-fold molar excess). Note that in panel A, 50-fold higher concentrations of segment-CS or segment-CL to segment-C-Bpa did not lead to full suppression of the segment-CS-Bpa photoadduct in Figure S10A. However, this effect has been seen by us (2) and others (3) upon introduction of bulky hydrophobic residues such as Trp or Bpa into the *N*-terminus of amphipathic CaM-binding peptides and suggested a positive correlation between hydrophobicity of *N*-terminal anchor positions and affinity for CaM binding. In line with the fact that this effect was not apparent when hydrophobic amino acids were introduced more *C*-terminally (3), we did not observe it with segment-C-Bpa. PA: photo-adduct of CaM and bMunc13-2 segment-C-Bpa/segment-CS-Bpa

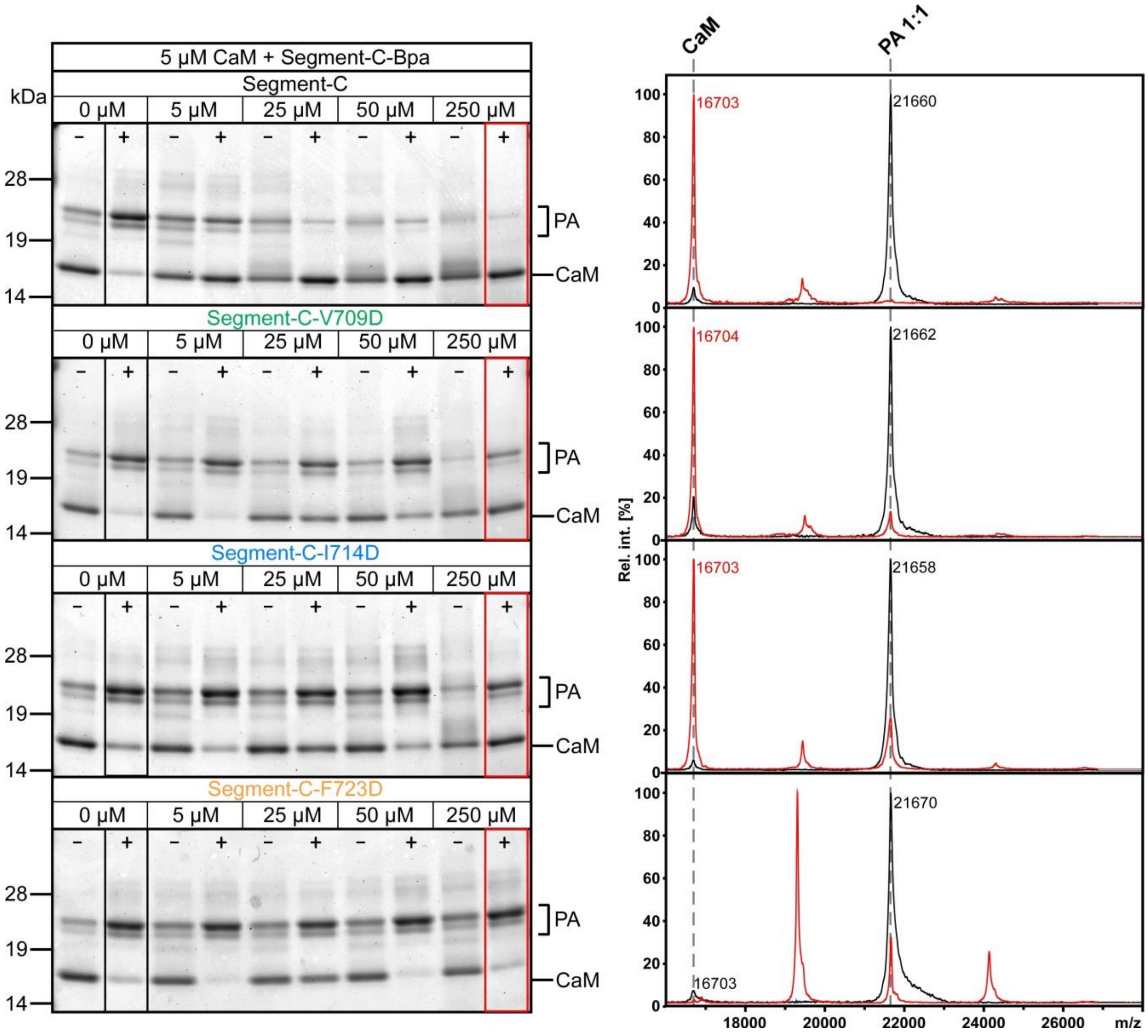

Figure S11: SDS-PAGE and MALDI-TOF-MS analysis of PAL-based competition assays with bMunc13-2 segment-C-Bpa and the segment-C variants. (-) sample without Ca^{2+}, (+) sample containing 200 µM Ca^{2+}. The left panels display the SDS-PAGE analysis; the right panels show the mass spectra of the formation of the CaM/bMunc13-2 complex. The black trace in the mass spectra refers to the samples boxed in black in the gel image (presence of Ca^{2+}, absence of competitor); the red trace in the mass spectra refers to the samples boxed in red in the gel image (presence of Ca^{2+}, presence of competitor in 50-fold molar excess). PA: photo-adduct of CaM and bMunc13-2 segment-C-Bpa.

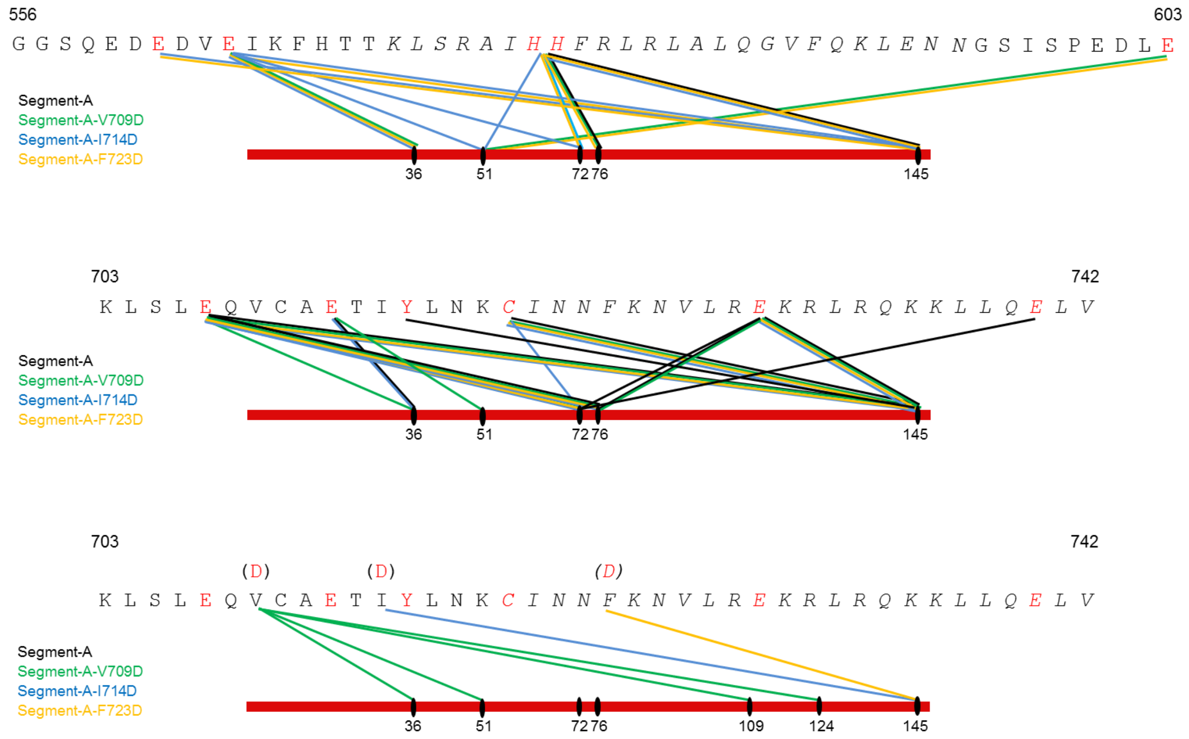

Figure S12: Photo-Met-cross-links within the CaM binding regions of bMunc13-2 mutants. The CaM binding regions and flanking amino acid sequences harboring the cross-linked amino acids are shown. The proposed CaM binding region is highlighted in italic letters. Cross-linked amino acids are colored in red. In the lower panel the respective cross-links including the exchanged amino acids (shown in brackets) are displayed. The cross-links are colored for each segment-A variant. Black: wildtype, green: V709D, blue: I714D and orange: F723D.

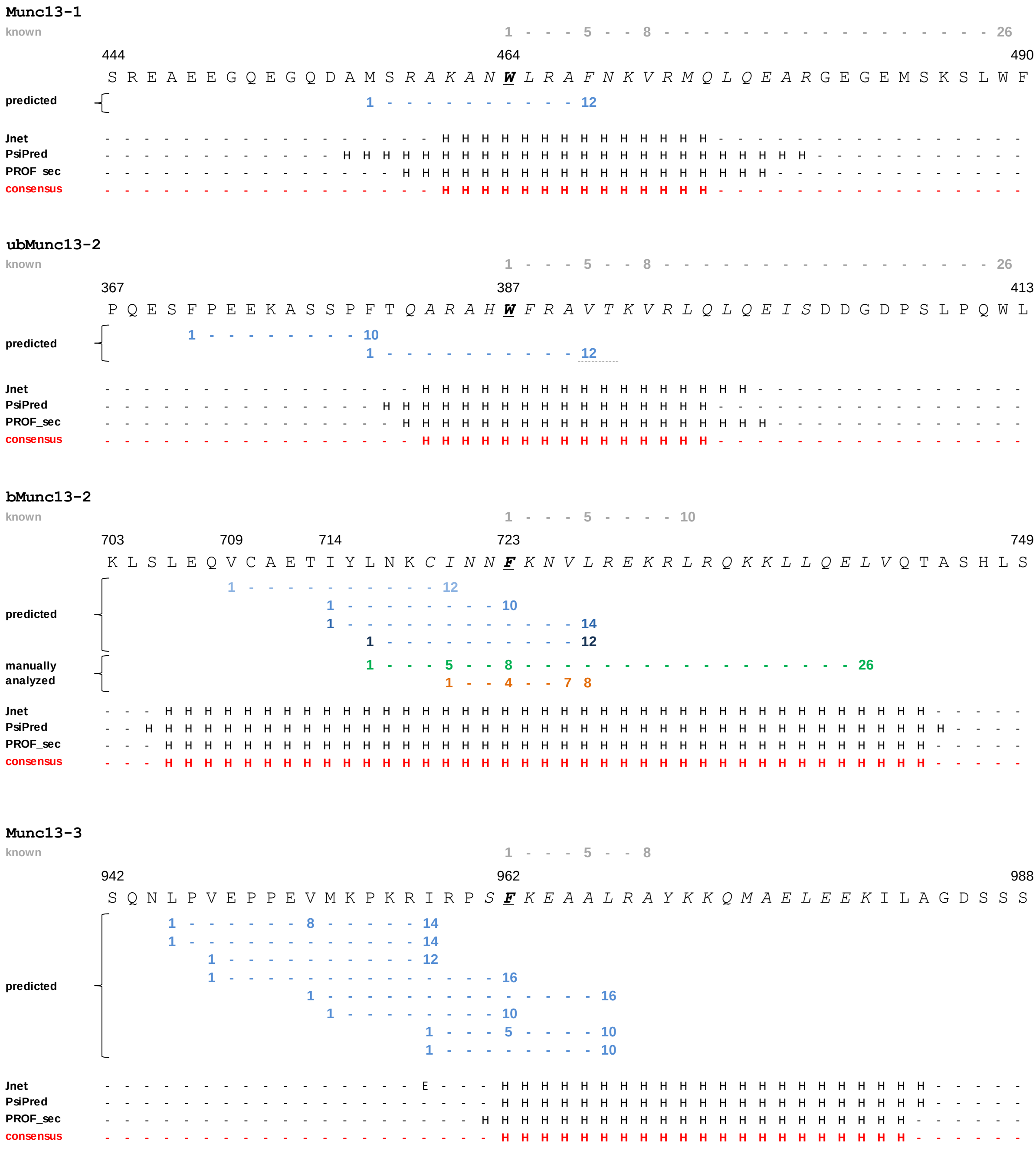

Figure S13: Analysis of possible CaM binding motifs and secondary structure prediction of CaM binding sites of the Munc13 isoforms. The prevailing amino acid sequences for the CaM binding regions of all four Munc13 isoforms corresponding to the CaM-binding site in bMunc13-2 are displayed. The known or proposed CaM binding regions are highlighted in italic. Possible binding motifs and secondary structure predictions are shown. Grey: known binding motif, blue: motifs identified with the CaM binding motif database, green/orange: manually analyzed CaM binding motif, H: helical region, E: sheet region, -: coiled region.

Protein	Species	Method	Sequence / prediction
Munc13-1	human		P R E D E E G Q E G Q D S M S R A K A N W L R A F N K V R M Q L Q E A R G E G E M S K S L W F
		Jnet	- - - - - - - - - - - - - - H H H H H H H H H H H H H H H H H H - - - - - - - - - - - - - - -
		PsiPred	- - - - - - - - - - - - - H H H H H H H H H H H H H H H H H H H H H H H - - - - - - - - - - -
		PROF_sec	- - - - - - - - - - - - - - - H H H H H H H H H H H H H H H H H - - - - - - - - - - - - - - -
		consensus	- - - - - - - - - - - - - - - H H H H H H H H H H H H H H H H H - - - - - - - - - - - - - - -
	rat		S R E A E E G Q E G Q D A M S R A K A N W L R A F N K V R M Q L Q E A R G E G E M S K S L W F
		Jnet	- - - - - - - - - - - - - - - - - H H H H H H H H H H H H H H - - - - - - - - - - - - - - - -
		PsiPred	- - - - - - - - - - - - H H H H H H H H H H H H H H H H H H H H H H H H - - - - - - - - - - -
		PROF_sec	- - - - - - - - - - - - - - - H H H H H H H H H H H H H H H H H H H - - - - - - - - - - - - -
		consensus	- - - - - - - - - - - - - - - - - H H H H H H H H H H H H H H - - - - - - - - - - - - - - - -
	zebrafish		I P P K A E Q E K K E A L P E R A K A N W L R L F N R V R L Q L Q E A R G E T P G L A S L F L
		Jnet	- - - - - - - - - - - - - - H H H H H H H H H H H H H H H H H H H H H - - - - - - - - - - - -
		PsiPred	- - - - H H H H H - - - - H H H H H H H H H H H H H H H H H H H H H H H - - - - - - - - - - -
		PROF_sec	- - - - - - - - - - - - - - - H H H H H H H H H H H H H H H H H H H H - - - - - - - - - - - -
		consensus	- - - - - - - - - - - - - - - H H H H H H H H H H H H H H H H H H H H - - - - - - - - - - - -
		overall	- - - - - - - - - - - - - - - - H H H H H H H H H H H H H H H - - - - - - - - - - - - - - - -
ubMunc13-2	human		P Q E S F P E E K A S S P F T Q A R A H W F R A V T K V R L Q L Q E I S D D G D P S L P Q W L
		Jnet	- - - - - - - - - - - - - - - - H H H H H H H H H H H H H H H H H - - - - - - - - - - - - - -
		PsiPred	- - - - - - - - - - - - - - H H H H H H H H H H H H H H H H H - - - - - - - - - - - - - - - -
		PROF_sec	- - - - - - - - - - - - - - - H H H H H H H H H H H H H H H H H H H - - - - - - - - - - - - -
		consensus	- - - - - - - - - - - - - - - - H H H H H H H H H H H H H H H - - - - - - - - - - - - - - - -
	rat		P Q E S F P E E K A S S P F T Q A R A H W F R A V T K V R L Q L Q E I S D D G D P S L P Q W L
		Jnet	- - - - - - - - - - - - - - - - H H H H H H H H H H H H H H H H H - - - - - - - - - - - - - -
		PsiPred	- - - - - - - - - - - - - - H H H H H H H H H H H H H H H H H - - - - - - - - - - - - - - - -
		PROF_sec	- - - - - - - - - - - - - - - H H H H H H H H H H H H H H H H H H H - - - - - - - - - - - - -
		consensus	- - - - - - - - - - - - - - - - H H H H H H H H H H H H H H H - - - - - - - - - - - - - - - -
	zebrafish		S P K S F K D D G L P L P F S P S R V R W L K A I N K V R V Q L K E G K E N G D N S R H A W V
		Jnet	- - - - - - - - - - - - - - - - H H H H H H H H H H H H H H H H - - - - - - - - - - - - - - -
		PsiPred	- - - - - - - - - - - - - - - H H H H H H H H H H H H H H H H H H H - - - - - - - - - - - - -
		PROF_sec	- - - - - - - - - - - - - - - H H H H H H H H H H H H H H H H H H H - - - - - - - - - - - - -
		consensus	- - - - - - - - - - - - - - - - H H H H H H H H H H H H H H H H - - - - - - - - - - - - - - -
		overall	- - - - - - - - - - - - - - - - H H H H H H H H H H H H H H H - - - - - - - - - - - - - - - -

Figure S14: Secondary structure prediction of CaM binding sites within Munc13-1 and ubMunc13-2 of human, rat and zebrafish. H: helical region, E: sheet region, -: coiled region

	**human**		**R**	**L**	**S**	**L**	**E**	**Q**	**V**	**C**	**T**	**E**	**T**	**V**	**Y**	**L**	**N**	**K**	**C**	**I**	**N**	**N**	**F**	**K**	**N**	**V**	**L**	**R**	**E**	**K**	**R**	**L**	**R**	**Q**	**K**	**K**	**L**	**L**	**H**	**E**	**L**	**V**	**Q**	**K**	**A**	**N**	**R**	**L**	**S**
		**Jnet**	-	-	-	H	H	H	H	H	H	H	H	H	H	H	H	H	H	H	H	H	H	H	H	H	H	H	H	H	H	H	H	H	H	H	H	H	H	H	H	H	H	H	H	H	-	-	-
		**PsiPred**	-	-	H	H	H	H	H	H	H	H	H	H	H	H	H	H	H	H	H	H	H	H	H	H	H	H	H	H	H	H	H	H	H	H	H	H	H	H	H	H	H	H	H	H	-	-	-
		**PROF_sec**	-	-	-	H	H	H	H	H	H	H	H	H	H	H	H	H	H	H	H	H	H	H	H	H	H	H	H	H	H	H	H	H	H	H	H	H	H	H	H	H	H	H	H				
		**consensus**	-	-	-	**H**	**H**	**H**	**H**	**H**	**H**	**H**	**H**	**H**	**H**	**H**	**H**	**H**	**H**	**H**	**H**	**H**	**H**	**H**	**H**	**H**	**H**	**H**	**H**	**H**	**H**	**H**	**H**	**H**	**H**	**H**	**H**	**H**	**H**	**H**	**H**	**H**	**H**	**H**	**H**	-	-	-	-
**bMunc13-2**	**rat**		**K**	**L**	**S**	**L**	**E**	**Q**	**V**	**C**	**A**	**E**	**T**	**I**	**Y**	**L**	**N**	**K**	**C**	**I**	**N**	**N**	**F**	**K**	**N**	**V**	**L**	**R**	**E**	**K**	**R**	**L**	**R**	**Q**	**K**	**K**	**L**	**L**	**Q**	**E**	**L**	**V**	**Q**	**T**	**A**	**S**	**H**	**L**	**S**
		**Jnet**	-	-	-	H	H	H	H	H	H	H	H	H	H	H	H	H	H	H	H	H	H	H	H	H	H	H	H	H	H	H	H	H	H	H	H	H	H	H	H	H	-	-	-	-	-
		**PsiPred**	-	-	H	H	H	H	H	H	H	H	H	H	H	H	H	H	H	H	H	H	H	H	H	H	H	H	H	H	H	H	H	H	H	H	H	H	H	H	H	H	H	-	-	-	-
		**PROF_sec**	-	-	-	H	H	H	H	H	H	H	H	H	H	H	H	H	H	H	H	H	H	H	H	H	H	H	H	H	H	H	H	H	H	H	H	H	H	H	H	H	-	-	-	-	-
		**consensus**	-	-	-	**H**	**H**	**H**	**H**	**H**	**H**	**H**	**H**	**H**	**H**	**H**	**H**	**H**	**H**	**H**	**H**	**H**	**H**	**H**	**H**	**H**	**H**	**H**	**H**	**H**	**H**	**H**	**H**	**H**	**H**	**H**	**H**	**H**	**H**	**H**	**H**	**H**	-	-	-	-	-
	**zebrafish**		**G**	**G**	**S**	**E**	**A**	**E**	**L**	**S**	**Q**	**R**	**N**	**A**	**C**	**R**	**L**	**K**	**C**	**L**	**R**	**S**	**F**	**Q**	**Q**	**I**	**L**	**R**	**E**	**K**	**R**	**E**	**S**	**R**	**K**	**Q**	**L**	**S**	**I**	**V**	**S**	**M**	**S**	**T**	**F**	**S**	**E**	**D**	**D**
		**Jnet**	-	-	-	-	-	H	H	H	H	H	H	H	H	H	H	H	H	H	H	H	H	H	H	H	H	H	H	H	H	H	H	H	H	H	H	H	H	H	-	-	-	-	-	E	-	-	-
		**PsiPred**	-	-	-	H	H	H	H	H	H	H	H	H	H	H	H	H	H	H	H	H	H	H	H	H	H	H	H	H	H	H	H	H	H	H	H	H	H	H	H	-	-	-	-	-	-	-	-
		**PROF_sec**	-	-	-	-	-	E	E	E	E	H	H	H	H	H	H	H	H	H	H	H	H	H	H	H	H	H	H	H	H	H	H	H	H	H	-	-	-	-	-	-	-	-	-	-	-	-	-
		**consensus**	-	-	-	-	-	-	-	-	-	**H**	**H**	**H**	**H**	**H**	**H**	**H**	**H**	**H**	**H**	**H**	**H**	**H**	**H**	**H**	**H**	**H**	**H**	**H**	**H**	**H**	**H**	**H**	**H**	**H**	-	-	-	-	-	-	-	-	-	-	-	-	-
		**overall**	-	-	-	-	-	-	-	-	-	**H**	**H**	**H**	**H**	**H**	**H**	**H**	**H**	**H**	**H**	**H**	**H**	**H**	**H**	**H**	**H**	**H**	**H**	**H**	**H**	**H**	**H**	**H**	**H**	**H**	-	-	-	-	-	-	-	-	-	-	-	-	-

	**human**		**N**	**Q**	**N**	**I**	**P**	**E**	**Q**	**P**	**V**	**E**	**I**	**T**	**K**	**P**	**K**	**R**	**I**	**R**	**P**	**S**	**F**	**K**	**E**	**A**	**A**	**L**	**R**	**A**	**Y**	**K**	**K**	**Q**	**M**	**A**	**E**	**L**	**E**	**E**	**K**	**I**	**L**	**A**	**G**	**D**	**S**	**S**	**S**
		**Jnet**	-	-	-	-	-	-	-	-	-	-	-	-	-	-	-	-	E	-	-	-	H	H	H	H	H	H	H	H	H	H	H	H	H	H	H	H	H	H	H	H	H	H	-	-	-	-	-
		**PsiPred**	-	-	-	-	-	-	-	-	-	-	-	-	-	-	-	-	-	-	-	-	H	H	H	H	H	H	H	H	H	H	H	H	H	H	H	H	H	H	H	H	H	-	-	-	-	-	-
		**PROF_sec**	-	-	-	-	-	-	-	-	-	-	-	-	-	-	-	-	-	-	-	-	H	H	H	H	H	H	H	H	H	H	H	H	H	H	H	H	H	H	H	H	H	H	-	-	-	-	H
		**consensus**	-	-	-	-	-	-	-	-	-	-	-	-	-	-	-	-	-	-	-	-	**H**	**H**	**H**	**H**	**H**	**H**	**H**	**H**	**H**	**H**	**H**	**H**	**H**	**H**	**H**	**H**	**H**	**H**	**H**	**H**	**H**	-	-	-	-	-	-
**Munc13-3**	**rat**		**S**	**Q**	**N**	**L**	**P**	**V**	**E**	**P**	**P**	**E**	**V**	**M**	**K**	**P**	**K**	**R**	**I**	**R**	**P**	**S**	**F**	**K**	**E**	**A**	**A**	**L**	**R**	**A**	**Y**	**K**	**K**	**Q**	**M**	**A**	**E**	**L**	**E**	**E**	**K**	**I**	**L**	**A**	**G**	**D**	**S**	**S**	**S**
		**Jnet**	-	-	-	-	-	-	-	-	-	-	-	-	-	-	-	-	E	-	-	-	H	H	H	H	H	H	H	H	H	H	H	H	H	H	H	H	H	H	H	H	H	H	-	-	-	-	-
		**PsiPred**	-	-	-	-	-	-	-	-	-	-	-	-	-	-	-	-	-	-	-	-	H	H	H	H	H	H	H	H	H	H	H	H	H	H	H	H	H	H	H	H	H	H	-	-	-	-	-
		**PROF_sec**	-	-	-	-	-	-	-	-	-	-	-	-	-	-	-	-	-	-	-	H	H	H	H	H	H	H	H	H	H	H	H	H	H	H	H	H	H	H	H	H	H	-	-	-	-	-	-
		**consensus**	-	-	-	-	-	-	-	-	-	-	-	-	-	-	-	-	-	-	-	-	**H**	**H**	**H**	**H**	**H**	**H**	**H**	**H**	**H**	**H**	**H**	**H**	**H**	**H**	**H**	**H**	**H**	**H**	**H**	**H**	**H**	-	-	-	-	-	-
	**zebrafish**		**K**	**N**	**V**	**T**	**E**	**V**	**P**	**V**	**Q**	**E**	**A**	**P**	**P**	**K**	**K**	**R**	**I**	**R**	**P**	**T**	**F**	**K**	**E**	**A**	**A**	**L**	**R**	**A**	**Y**	**R**	**K**	**Q**	**M**	**A**	**E**	**L**	**E**	**Q**	**Q**	**I**	**L**	**A**	**G**	**D**	**S**	**T**	**A**
		**Jnet**	-	-	-	-	-	-	-	-	-	-	-	-	-	-	-	-	E	-	-	-	H	H	H	H	H	H	H	H	H	H	H	H	H	H	H	H	H	H	H	H	H	H	-	-	-	-	-
		**PsiPred**	-	-	-	-	-	-	-	-	-	-	-	-	-	-	-	-	-	-	-	-	H	H	H	H	H	H	H	H	H	H	H	H	H	H	H	H	H	H	H	H	H	H	-	-	-	-	-
		**PROF_sec**	-	-	-	-	-	-	-	-	-	-	-	-	-	-	-	-	-	-	-	-	H	H	H	H	H	H	H	H	H	H	H	H	H	H	H	H	H	H	H	H	H	-	-	-	-	-	-
		**consensus**	-	-	-	-	-	-	-	-	-	-	-	-	-	-	-	-	-	-	-	-	**H**	**H**	**H**	**H**	**H**	**H**	**H**	**H**	**H**	**H**	**H**	**H**	**H**	**H**	**H**	**H**	**H**	**H**	**H**	**H**	**H**	-	-	-	-	-	-
		**overall**	-	-	-	-	-	-	-	-	-	-	-	-	-	-	-	-	-	-	-	-	**H**	**H**	**H**	**H**	**H**	**H**	**H**	**H**	**H**	**H**	**H**	**H**	**H**	**H**	**H**	**H**	**H**	**H**	**H**	**H**	**H**	-	-	-	-	-	-

Figure S15: Secondary structure prediction of CaM binding sites within bMunc13-2 and Munc13-3 of human, rat and zebrafish.

Supplementary References

1. Piotrowski, C., Ihling, C. H., and Sinz, A. (2015) Extending the cross-linking/mass spectrometry strategy: Facile incorporation of photo-activatable amino acids into the model protein calmodulin in Escherichia coli cells. *Methods* **89**, 121-127
2. Dimova, K., Kawabe, H., Betz, A., Brose, N., and Jahn, O. (2006) Characterization of the Munc13-calmodulin interaction by photoaffinity labeling. *Biochim. Biophys. Acta* **1763**, 1256-1265
3. O'Neil, K.T., Erickson-Vlitanen, S., and DeGrado, W.F. (1989) Photolabeling of calmodulin with basic, amphiphilic alpha-helical peptides containing benzoylphenylalanine. *J. Biol. Chem.* **264**, 14571-14578.
