# Supplementary material for: Delineating the Molecular Basis of the Calmodulin–bMunc13-2 Interaction by Cross-Linking/Mass Spectrometry—Evidence for a Novel CaM Binding Motif in bMunc13-2
Source: Cells. 2020 Jan 7;9(1):136. doi: 10.3390/cells9010136 (PMC7017353; doi:10.3390/cells9010136)
Supplement: Supplementary file 1 [file cells-09-00136-s001.zip › Cells-661696_Supplementary_files/Cells-661696_III-SupplMethods.pdf]

SUPPLEMENTARY MATERIAL III

Supplementary Methods: Protocol capture page for modeling approach

Delineating the Molecular Basis of the Calmodulin/bMunc13-2 Interaction by Cross-linking/Mass Spectrometry – Evidence for a Novel CaM Binding Motif in bMunc13-2

Christine Piotrowski[1], Rocco Moretti[2], Christian H. Ihling[1], André Haedicke[3,†], Thomas Liepold[4], Noa Lipstein[5], Jens Meiler[2], Olaf Jahn[4,*], Andrea Sinz[1,*]

[1] Department of Pharmaceutical Chemistry and Bioanalytics, Institute of Pharmacy, Charles Tanford Protein Center, Martin Luther University Halle-Wittenberg, D-06120 Halle/Saale, Germany;

[2] Center for Structural Biology, Department of Chemistry, Vanderbilt University, Nashville, TN 37221, USA

[3] Biophysical Chemistry, Institute of Chemistry, Martin Luther University Halle-Wittenberg, D-06120 Halle/Saale, Germany

[4] Proteomics Group, Max Planck Institute of Experimental Medicine, D-37075 Göttingen, Germany

[5] Department of Molecular Neurobiology, Max Planck Institute of Experimental Medicine, D-37075 Göttingen, Germany

* Correspondence: jahn@em.mpg.de (O.J.); andrea.sinz@pharmazie.uni-halle.de (A.S.); Tel.: +49-551-3899-313 (O.J.); +49-345-5525170 (A.S.)

† Present address: Serumwerk Bernburg AG, D-06406 Bernburg, Germany.

Protocol Capture for Piotrowski et al (2019): Delineating the Molecular Basis of the Calmodulin/bMunc13-2 Interaction by Cross-linking/Mass Spectrometry – Evidence for a Novel CaM Binding Motif in bMunc13-2

- Overview
- Background
- Protocol
  - Environment
  - Creating a starting structure for FlexPepDock application
  - Dock bMunc13-2 peptide into Calmodulin
  - Filtering and refinement of the generated structures

Overview

This is the protocol capture page for modeling of the interaction of calmodulin with a bMunc13-2 peptide (named segment-C) as described in "Delineating the Molecular Basis of the Calmodulin/ bMunc13-2 Interaction by Cross-linking/Mass Spectrometry – Evidence for a Novel CaM Binding Motif in bMunc13-2" by Christine Piotrowski, Rocco Moretti, Christian H. Ihling, André Haedicke, Noa Lipstein, Jens Meiler, Olaf Jahn and Andrea Sinz.

Background

Exploring the interactions between the Ca2+ binding protein calmodulin (CaM) and its target proteins remains a challenging task. Members of the Munc13 protein family, playing an essential role in short-term synaptic plasticity, are prominent CaM targets (Lipstein, 2017). In this study, we focus on the brain bMunc13-2 isoform as strong changes in signal transduction were observed when selected amino acids in bMunc13-2 were mutated or deleted or bMunc13-2 was knocked out (Varoqueaux, 2002; Lipstein, 2012). The CaM/ bMunc13-2 interaction has so far been studied on the molecular level only in peptide studies revealing a similar binding behavior compared to the other three Munc13 isoforms, Munc13-1, ubMunc13-2, and Munc13-3 (Lipstein, 2012). Cross-linking data were generated with a segment of bMunc13-2 (aa 367-780) containing the binding site for CaM, which should be used for modeling of the CaM/bMunc13-2 interaction. Most of the cross-links were observed in the region of aa 704-742 of bMunc13-2 whereas the respective sequence (aa 704-742, named segment-C) was applied for the modeling process. Segment-C was docked into a CaM structure derived from PDB-Code 2O60 by the Rosetta FlexPepDock application. Interestingly, the generated models displayed a kink within the helical structure of segment-C allowing a closed conformation of CaM while interacting with *C*-terminally and *N*-terminally located amino acids of bMunc13-2 segment-C. This conformation is not consistent with previous conformations observed for short or *C*-terminally extended peptides of Munc13-1, ubMunc13-2, bMunc13-2 and Munc13-3 and revealed a binding mode of bMunc13-2 differing from the remaining isoforms.

Protocol

Creating a starting structure for FlexPepDock application

First, bMunc13-2 segment-C (aa 704-742, `LSLEQVCAETIYLNKCINNFKNVLREKRLRQKKLLQELV`) was built in a preliminary helical structure to place it manually into a CaM structure, which was derived from the structural model of a peptide from the NO-synthase interacting with CaM in a closed conformation (PDB-Code: 2O60). This CaM structure was selected because the amino acid sequence of the NO-peptide displayed a good similarity to the bMunc13-2 segment-C in an alignment of the amino acid sequences, especially in the predicted anchor amino acids (green frame).

```
bMunc13-2 segment-C   LSLEQVCAETIYLNKCINNFKNVLREKRLRQKKLLQELV
NOS-peptide           ----------------AIGFKKLAEAVKFSAK-LMGQ-
                                         **::.  .  ::  * *: :
```

Step	Text	Commands	Comment
**1A.** Generate a helical structure for bMunc13-2 segment-C	For the amino acid sequence of bMunc13-2 segment-C a helical structure was generated by the Rosetta BuildPeptide application.	$rosettapath/main/source/bin/BuildPeptide.default.linuxgccrelease -in:file:fasta CINNFK.fasta -out:file:o CINNFK.pdb -helix	**Input file:** CINNFK.fasta<br><br>**Output file:** helix_CINNFK.pdb

**1B.** Identify a structure for CaM	Structures of CaM in complex with different peptides and proteins were manually inspected to fit to the bMunc13-2 segment-C sequence.	Amino acid sequence alignment on webpage: https://www.ebi.ac.uk/Tools/msa/clustalo/.	
**1C.** Generate input structure for FlexPepDock Application	The generated helical structure of bMunc13-2 segment-C was inserted manually into the CaM structure derived from PDB-file 2O60.	The helical structure of the peptide was placed in an antiparallel orientation and a central position into CaM. The structure was moved with the help of the mouse using the editing mode in Pymol.	**Input file:** helix_CINNFK.pdb 2O60.pdb<br><br>**Out file:** 2O60_CINNFK_peptide.pdb

Dock bMunc13-2 peptide into Calmodulin

Step	Text	Commands	Comment
**2A.** Set constraints for CaM binding site	The binding site was predicted to be identical to the remaining Munc13-isoforms. Therefore, distance restraints were set to have the correct location of the binding site. Distances were obtained from PDB-files: 2KDU and 2O60.<br>2O60 was chosen as the peptide of the NOS synthetase was highly similar to the bMunc13-2 segment-C sequence.	Measure distances between the C_α-atoms of the respective amino acids listed in the output file in Pymol with the “Wizard measurement” function.	**Output file:** constraints_F1_L5.cst
**2B:** Generate fragment files	For the modeling and docking of segment-C, fragments for building the secondary structure have to be generated.	/dors/meilerlab/apps/scripts/rosetta_tools/fragmentpicker_runss C $rosettapath/demos/protocol_capture/flex_pep_dock_abinitio/scripts/frags/ CINNFK_frags.200.3mers 148 > CINNFK_frags.3mers.offset	**Input files:** CINNFK.fasta fragment_picker_quota.options<br>**Output files:** CINNFK_frags.3mers.offset CINNFK_frags.5mers.offset CINNFK_frags.9mers.offset
**2C.** Run FlexPepDock	Segment-C is built and docked into CaM	#!/bin/tcsh<br>foreach a (`/usr/bin/seq 1 1 5`)<br>echo $a<br>$rosettapath/main/source/bin/FlexPepDocking.linuxgccrelease<br>-database<br>/dors/meilerlab/apps/rosetta/rosetta_2015.31.58019/main/database/ @flags<br>-nstruct 500 -out:file:silent decoys_$a.silent -scorefile score_$a.sc &>><br>Loop_fixed_F1_L5_without_constraints_$a.log<br>end	**Input file:** flags CINNFK_frags.3mers.offset CINNFK_frags.5mers.offset CINNFK_frags.9mers.offset<br><br>**Output files:** decoys_2o60_loop_fixed_f1_L5.silent

		Combine all files into one:<br>$rosettapath/main/source/bin/combine_silent.default.linuxgccrelease<br>-in:file:silent decoys_*.silent -out:file:silent decoys_2o60_loop_fixed_f1_L5.silent	10 000 models generated

Filtering and refinement of the generated structures

Step	Text	Commands	Comment
**3A.** Relax all models	Models were subjected to the relax application to get non-forced structures for subsequent filtering with the cross-linking restraints.	$rosettapath/main/source/bin/relax.linuxgccrelease -in:file:s decoys_2o60_loop_fixed_f1_L5.silent -out:file:silent decoys_2o60_loop_fixed_f1_L5_relax.silent	**Input file:**<br>decoys_2o60_loop_fixed_f1_L5.silent<br><br>**Output file:**<br>decoys_2o60_loop_fixed_f1_L5_relax.silent
**3B.** Apply cross-linking restraints	Rescore models with cross-linking restraints<br>- KofN files were used as not all cross-links can fit one conformation.	#!/bin/tcsh<br>foreach a (`/usr/bin/seq 5 1 7)<br>echo $a<br>$rosettapath/main/source/bin/score_jd2.linuxgccrelease<br>-in:file:silent decoys_2o60_loop_fixed_f1_L5_relax.silent<br>-constraints:cst_fa_file constraints_XL_KofN_new_$a.txt -constraints:cst_fa_weight 10<br>-constraints:cst_file constraints_XL_KofN_new_$a.txt -constraints:cst_weight 10<br>-out:file:score_only score_fixed_rescore_KofN_$a.sc<br>end<br><br>Remove "_0001" from names of generated structures.	**Input file:**<br>decoys_2o60_loop_fixed_f1_L5.silent<br>constraints_XL_KofN_new_5.txt<br>constraints_XL_KofN_new_6.txt<br>constraints_XL_KofN_new_7.txt<br><br>**Output files:**<br>score_fixed_rescore_KofN_5.sc<br>score_fixed_rescore_KofN_6.sc<br>score_fixed_rescore_KofN_7.sc<br>and respective pdb files in folders KofN_5 to KofN_7
**3C.** Select best scored models	Select best models which fit to 5, 6 and 7 cross-links by atom_pair restraints	#!/bin/tcsh<br>foreach a (`/usr/bin/seq 5 1 7`)<br>echo $a<br>/home/christine/Dokumente/modeling_new/cluster.sh 500 3 score_fixed_rescore_KofN_$a.sc 2o60_CINNFK_peptide.pdb decoys_2o60_loop_fixed_f1_L5.silent atom_pair_constraint<br><br>mkdir KofN_$a<br>mv c.*.pdb KofN_$a/<br>mv clog KofN_$a<br>mv clusters_by_atom_pair_constraint.txt KofN_$a<br><br>end	**Input files:**<br>cluster.sh<br>score_fixed_rescore_KofN_5.sc<br>score_fixed_rescore_KofN_6.sc<br>score_fixed_rescore_KofN_7.sc

		Join best structures in cluster with the join linux command.	**Output file:**<br>join_cluster_5_6_7_list.txt<br>pdb files of respective models<br>(181 models identified)
**3D.** Rescoring	Rescore models with most prominent cross-link	#!/bin/tcsh<br>foreach a (`/usr/bin/seq 5 1 7)<br>echo $a<br>/$rosettapath/main/source/bin/score_jd2.linuxgccrelease<br>-in:file:s *.pdb<br>-constraints:cst_fa_file constraints_XL_KofN_new_$a.txt -constraints:cst_fa_weight 10<br>-constraints:cst_file constraints_XL_KofN_new_$a.txt -constraints:cst_weight 10<br>-out:file:score_only score_fixed_rescore_KofN_$a.sc<br>end	**Input file:**<br>pdb files of models<br>onstraints_XL_76-4_145-16_sd3.txt<br><br>**Output files:**<br>rescore_relax_reasonable_76-4_145_16.sc<br>rescore_relax_reasonable_KofN7.sc
**3E.** Relax	Relaxation without any applied restraints to prevent forced structures which are only in the correct position due to the distance restraints.	$rosettapath/main/source/bin/relax.linuxgccrelease -in:file:s *.pdb	**Input and output files:**<br>pdb files of respective models
**3F.** Improved filtering of selected models	Rescore 181 models with KofN_7 and most prominent cross-links and cluster by atom_pair_constraint	$rosettapath/main/source/bin/score_jd2.linuxgccrelease -in:file:s *.pdb -constraints:cst_fa_file constraints_XL_76-4_145-16_sd2.txt -constraints:cst_fa_weight 10 -constraints:cst_file constraints_XL_76-4_145-16_sd2.txt -constraints:cst_weight 10 -out:file:score_only rescore_relax_reasonable_76-4_145_16.sc<br>$rosettapath/main/source/bin/score_jd2.linuxgccrelease -in:file:s *.pdb -constraints:cst_fa_file constraints_XL_ KofN_new_7.txt -constraints:cst_fa_weight 10 -constraints:cst_file constraints_XL KofN_new_7.txt -constraints:cst_weight 10 -out:file:score_only rescore_relax_reasonable_ KofN_new_7.sc<br><br>Plot atom_pair_constraints to total score and compare models at the pareto optimum between the two datafiles (KofN0/ and two most prominent cross-links)	**Input files:**<br>pdb files of respective models<br>constraints_XL_76-4_145-16_sd2.txt<br>constraints_XL_KofN_new_7.txt<br><br>**Output files:**<br>rescore_relax_reasonable_76-4_145_16.sc<br>rescore_relax_reasonable_KofN7.sc<br><br>Four models identified which overlap between both filtering approaches:<br>loop_fixed_F1_L5_without_XL_restraints_0071_8<br>loop_fixed_F1_L5_without_XL_restraints_0331_10<br>loop_fixed_F1_L5_without_XL_restraints_0334_8<br>loop_fixed_F1_L5_without_XL_restraints_0449_18

**3F.** Relax with cross-linking restraints	Relax the models with constraint files leading to two possible conformations.<br>Two constraint files were generated:<br>1) cross-links pointing to an interaction of bMunc13-2 segment-C with the C-terminal part of CaM<br>2) cross-links pointing to an interaction of bMunc13-2 segment-C with the central part of CaM	$rosettapath/main/source/bin/relax.linuxgccrelease -in:file:s loop_fixed_F1_L5_without_restraints* -constraints:cst_fa_file constraints_XL_145.txt -constraints:cst_fa_weight 10 -constraints:cst_file constraints_XL_145.txt -constraints:cst_weight 10 -nstruct 10<br><br>$rosettapath/main/source/bin/relax.linuxgccrelease -in:file:s loop_fixed_F1_L5_without_restraints* -constraints:cst_fa_file constraints_XL_76.txt -constraints:cst_fa_weight 10 -constraints:cst_file constraints_XL_76.txt -constraints:cst_weight 10 -nstruct 10	**Input file:**<br>constraints_XL_76.txt<br>constraints_XL_145.txt<br>loop_fixed_F1_L5_without_XL_restraints_0071_8.pdb<br>loop_fixed_F1_L5_without_XL_restraints_0331_10.pdb<br>loop_fixed_F1_L5_without_XL_restraints_0334_8.pdb<br>loop_fixed_F1_L5_without_XL_restraints_0449_18.pdb<br>**Output file:**<br>pdb files of respective models
**3G.** Relax without any restraints	Second relaxation without any applied restraints to prevent forced structures which are only in the correct position due to the distance restraints.	$rosettapath/main/source/bin/relax.linuxgccrelease -in:file:s loop_fixed_F1_L5_without_restraints*	**Input and output files:**<br>pdb files from step 3F
**3H.** Second refinement	Repeat step 3F and 3G to refine the models with lower sd value for the cross-linking restraints	$rosettapath/main/source/bin/relax.linuxgccrelease -in:file:s loop_fixed_F1_L5_without_restraints* -constraints:cst_fa_file constraints_XL_145.txt -constraints:cst_fa_weight 10 -constraints:cst_file constraints_XL_145.txt -constraints:cst_weight 10 -nstruct 10<br><br>$rosettapath/main/source/bin/relax.linuxgccrelease -in:file:s loop_fixed_F1_L5_without_restraints* -constraints:cst_fa_file constraints_XL_76.txt -constraints:cst_fa_weight 10 -constraints:cst_file constraints_XL_76.txt -constraints:cst_weight 10 -nstruct 10	**Input file:**<br>constraints_XL_76_sd05.txt<br>constraints_XL_145_sd05.txt<br>pdb files from step 3G<br>**Output files:**<br>pdb files of respective models
**3I.** Select best model	Manual inspection of the models in Pymol.	The models were inspected in terms of distances observed for the applied cross-links and orientation of the cross-linked amino acids to each other.	**Final models:**<br>loop_fixed_F1_L5_without_XL_restraints_0449_18_modelA<br>loop_fixed_F1_L5_without_XL_restraints_0449_18_modelB

Literature

Lipstein, 2012 — Lipstein, N., et al., Nonconserved Ca2+/Calmodulin Binding Sites in Munc13s Differentially Control Synaptic Short-Term Plasticity. Mol. Cell. Biol. 2012. 32(22): p. 4628-4641.

Lipstein, 2017 — Lipstein, N., et al., Presynaptic Calmodulin targets: lessons from structural proteomics. Expert Rev. Proteomics 2017. 14(3): p. 223-242.

Varoqueaux, 2002 — Varoqueaux, F., et al., Total arrest of spontaneous and evoked synaptic transmission but normal synaptogenesis in the absence of Munc13-mediated vesicle priming. Proc. Natl. Acad. Sci. USA 2002. 99(13): p. 9037-9042.
