# Supplementary material for: Delineating the Molecular Basis of the Calmodulin–bMunc13-2 Interaction by Cross-Linking/Mass Spectrometry—Evidence for a Novel CaM Binding Motif in bMunc13-2
Source: Cells. 2020 Jan 7;9(1):136. doi: 10.3390/cells9010136 (PMC7017353; doi:10.3390/cells9010136)
Supplement: Supplementary file 1 [file cells-09-00136-s001.zip › Cells-661696_Supplementary_files/Cells_661696_II-SupplTabs.pdf]

SUPPLEMENTARY MATERIAL II

Supplementary Tables

Delineating the Molecular Basis of the Calmodulin/bMunc13-2 Interaction by Cross-linking/Mass Spectrometry – Evidence for a Novel CaM Binding Motif in bMunc13-2

Christine Piotrowski[1], Rocco Moretti[2], Christian H. Ihling[1], André Haedicke[3,†], Thomas Liepold[4], Noa Lipstein[5], Jens Meiler[2], Olaf Jahn[4,*], Andrea Sinz[1,*]

[1] Department of Pharmaceutical Chemistry and Bioanalytics, Institute of Pharmacy, Charles Tanford Protein Center, Martin Luther University Halle-Wittenberg, D-06120 Halle/Saale, Germany;

[2] Center for Structural Biology, Department of Chemistry, Vanderbilt University, Nashville, TN 37221, USA

[3] Biophysical Chemistry, Institute of Chemistry, Martin Luther University Halle-Wittenberg, D-06120 Halle/Saale, Germany

[4] Proteomics Group, Max Planck Institute of Experimental Medicine, D-37075 Göttingen, Germany

[5] Department of Molecular Neurobiology, Max Planck Institute of Experimental Medicine, D-37075 Göttingen, Germany

*Correspondence: jahn@em.mpg.de (O.J.); andrea.sinz@pharmazie.uni-halle.de (A.S.); Tel.: +49-551-3899-313 (O.J.); +49-345-5525170 (A.S.)

†Present address: Serumwerk Bernburg AG, D-06406 Bernburg, Germany.

Table S1: Photo-Met cross-links with CaM and bMunc13-2 segment-A at different Ca^{2+} concentrations. x denotes photo-Met.

**Cross-linked amino acids CaM**	**Cross-linked amino acids bMunc13-2**	**Ca^{2+} concentration**				
		750 nM	2.5 µM	45 µM	300 µM	1 mM
x36	D615					✓
x36	E712	✓	✓	✓	✓	✓
x71/x72	E707	✓	✓	✓	✓	✓
x71/x72	E740					✓
x76	H578	✓	✓	✓	✓	✓
x76	E707	✓	✓	✓	✓	✓
x76	E729		✓			✓
x144/x145	H578/H579	✓	✓	✓	✓	✓
x144/x145	E707					✓
x144/x145	Y715	✓	✓	✓		✓
x144/x145	C719	✓	✓	✓	✓	✓
x144/x145	E729	✓	✓	✓	✓	✓

Table S2: Summary of intermolecular cross-links between CaM and bMunc13-2 segment-A. x denotes photo-Met, B: carbamidomethylated Cys and m: oxidized Met. Small letters indicate 15N-labeling. CaM is indicated without the initial Met, bMunc13-2 with the initial Met.

Cross-Linker	Cross-linked amino acids CaM	Cross-linked amino acids bMunc13-2	Calculated molecular weight $[M+H]^+$	Charge state	Amino acid sequence CaM	Amino acid sequence bMunc13-2
Photo-Met	x36	D615	1146.623	3	$_{32}$LGTVxR$_{37}$	$_{613}$NSDR$_{616}$
	x36	E712	2436.317	3	$_{32}$LGTVxR$_{37}$	$_{704}$LSLEQVBAETIYLNK$_{718}$
	x36	E712	2565.359	3	$_{31}$ELGTVxR$_{37}$	$_{704}$LSLEQVBAETIYLNK$_{718}$
	x71/x72	E707	1896.956	2, 3	$_{68}$FLTxMAR$_{74}$ / $_{68}$FLTMxAR$_{74}$	$_{704}$LSLEQVBAE$_{712}$
	x71/x72	E707	1905.929	2	$_{68}$fltxmar$_{74}$ / $_{68}$fltmxar$_{74}$	$_{704}$LSLEQVBAE$_{712}$
	x71/x72	E707	1912.951	2, 3	$_{68}$FLTxmAR$_{74}$ / $_{68}$FLTmxAR$_{74}$	$_{704}$LSLEQVBAE$_{712}$
	x71/x72	E729	1296.709	3	$_{68}$FLTxmAR$_{74}$ / $_{68}$FLTmxAR$_{74}$	$_{729}$EKR$_{731}$
	x71/x72	E740	3257.709	4	$_{68}$FLTxmAR$_{74}$ / $_{68}$FLTmxAR$_{74}$	$_{737}$LLQELVQTASHLSVEDIPSEGK$_{758}$
	x71/x72	E740	3413.810	5	$_{68}$FLTxmAR$_{74}$ / $_{68}$FLTmxAR$_{74}$	$_{737}$LLQELVQTASHLSVEDIPSEGKR$_{759}$
	x71/x72	E740	3526.857	4	$_{68}$FLTxMAR$_{74}$ / $_{68}$FLTMxAR$_{74}$	$_{737}$LLQELVQTASHLSVEDIPSEGKRE$_{760}$
	x76	H578	1165.695	3	$_{75}$KxK$_{77}$	$_{576}$AIHHFR$_{561}$
	x76	H578	2369.184	5	$_{75}$KxKDTDSEEEIRE$_{87}$	$_{576}$AIHHFR$_{561}$
	x76	H578	2386.134	4, 5	$_{75}$kxkdtdseeeire$_{87}$	$_{576}$AIHHFR$_{561}$
	x76	E707	1433.767	3	$_{75}$KxK$_{77}$	$_{704}$LSLEQVBAE$_{712}$
	x76	E707	2110.933	3	$_{76}$xKDTDSEEE$_{87}$	$_{704}$LSLEQVBAE$_{712}$
	x76	E707	2239.028	3, 4	$_{75}$KxKDTDSEEE$_{84}$	$_{704}$LSLEQVBAE$_{712}$
	x76	E707	2249.996	3	$_{75}$kxkdtdseee$_{84}$	$_{704}$LSLEQVBAE$_{712}$
	x76	E707	2509.161	3	$_{76}$xKDTDSEEEIRE$_{87}$	$_{704}$LSLEQVBAE$_{712}$
	x76	E707	2524.117	3	$_{76}$xkdtdseeeire$_{87}$	$_{704}$LSLEQVBAE$_{712}$
	x76	E707	2637.256	3, 4, 5	$_{75}$KxKDTDSEEEIRE$_{87}$	$_{704}$LSLEQVBAE$_{712}$
	x76	E707	2654.206	4, 5	$_{75}$kxkdtdseeeire$_{87}$	$_{704}$LSLEQVBAE$_{712}$
	x76	E729	2021.015	3	$_{75}$KxKDTDSEEEIRE$_{87}$	$_{729}$EKR$_{731}$
	x76	E729	2037.967	3	$_{75}$kxkdtdseeeire$_{87}$	$_{729}$EKR$_{731}$
	x144/x145	E707	1982.993	3	$_{141}$FVQxMTAK$_{148}$ / $_{141}$FVQMxTAK$_{148}$	$_{704}$LSLEQVBAE$_{712}$
	x144/x145	E707	1998.987	3	$_{141}$FVQxmTAK$_{148}$ / $_{141}$FVQmxTAK$_{148}$	$_{704}$LSLEQVBAE$_{712}$
	x144/x145	H578/H579	1714.921	3	$_{1141}$FVQxMTAK$_{148}$ / $_{141}$FVQMxTAK$_{148}$	$_{576}$AIHHFR$_{561}$
	x144/x145	H578/H579	1723.894	3	$_{1141}$fvqxmtak$_{148}$ / $_{141}$fvqmxtak$_{148}$	$_{576}$AIHHFR$_{561}$
	x144/x145	H578/H579	1730.916	4	$_{141}$FVQxmTAK$_{148}$ / $_{141}$FVQmxTAK$_{148}$	$_{576}$AIHHFR$_{561}$
	x145	Y715	1685.929	3	$_{141}$FVQMxTAK$_{148}$	$_{713}$TIYLNK$_{718}$
	x144/x145	Y715	1928.056	3, 4	$_{141}$FVQMxTAKLE$_{150}$ / $_{141}$FVQMxTAKLE$_{150}$	$_{713}$TIYLNK$_{718}$
	x144/x145	Y715	1939.024	4	$_{141}$fvqmxtakle$_{150}$ / $_{141}$fvqmxtakle$_{150}$	$_{3}$TIYLNK$_{718}$

Table S2 continued

Cross-Linker	Cross-linked amino acids CaM	Cross-linked amino acids bMunc13-2	Calculated molecular weight $[M+H]^+$	Charge state	Amino acid sequence CaM	Amino acid sequence bMunc13-2
Photo-Met	x144/x145	C719	1672.855	3, 4	$_{141}$FVQxMTAK$_{148}$ / $_{141}$FVQMxTAK$_{148}$	$_{719}$CINNFK$_{724}$
	x144/x145	C719	1681.828	3, 4	$_{141}$fvqxmtak$_{148}$ / $_{141}$fvqmxtak$_{148}$	$_{719}$CINNFK$_{724}$
	x144/x145	C719	1688.850	3, 4	$_{141}$FVQxmTAK$_{148}$ / $_{141}$FVQmxTAK$_{148}$	$_{719}$CINNFK$_{724}$
	x144/x145	C719	1914.982	4	$_{141}$FVQMxTAKLE$_{150}$ / $_{141}$FVQMxTAKLE$_{150}$	$_{719}$CINNFK$_{724}$
	x144/x145	C719	3223.456	3	$_{126}$EADIDGDGQVNYEEFVQxmTAK$_{148}$ / $_{126}$EADIDGDGQVNYEEFVQmxTAK$_{148}$	$_{719}$CINNFK$_{724}$
	x144/x145	E729	1366.751	3	$_{141}$FVQxMTAK$_{148}$ / $_{141}$FVQMxTAK$_{148}$	$_{729}$EKR$_{731}$
	x144/x145	E729	1619.845	3	$_{141}$fvqxmtakle$_{150}$ / $_{141}$fvqmxtakle$_{150}$	$_{729}$EKR$_{731}$
DSBU	K75	K735	1809.853	3	$_{75}$KMKDTDSEEE$_{84}$	$_{734}$QKK$_{736}$
s-GMBS	K14	C428	1267.515	3	$_{13}$FKE$_{15}$	$_{425}$EEDCGK$_{430}$
	K31	C428	1880.770	3	$_{23}$DGDGTITTKE$_{32}$	$_{425}$EEDCGK$_{430}$
	K75	C453	1675.814	3, 4	$_{75}$KMK$_{77}$	$_{449}$LQSDCNNAIK$_{458}$

Table S3: Intramolecular cross-links identified in segment-A (cross-linkers BS3 and DSBU). Amine (in Lys) and hydroxy groups (in Ser, Thr, Tyr) were considered as reaction sites for NHS esters. Ca^{2+} concentrations are indicated.

		**Monomer**					**Heterodimer**	
		**DSBU**			**BS^3**		**DSBU**	
		75 nM	750 nM	1 mM	75 nM	1 mM	750 nM	1 mM
S378	K386	✓						
K380	K386		✓		✓		✓	✓
K380	K392						✓	✓
K380	K473	✓	✓	✓				✓
K380	K502			✓				
K380	K735						✓	
K380	K736			✓				
S382	K473			✓				
K386	K473			✓				
K386	K502			✓				✓
K392	K502							✓
S418	K473			✓				
K435	K502		✓					
K442	S451		✓	✓			✓	
K442	K473	✓	✓	✓				✓
K442	K502			✓			✓	
K448	K473		✓					
K448	K502		✓					
S466	K473			✓				
K470	K502			✓				
K473	K502	✓	✓	✓	✓	✓	✓	✓
K473	K572			✓	✓			
K473	K730	✓		✓			✓	
K473	K735		✓					✓
K473	K736			✓	✓			
K493	K502						✓	✓
K502	K550							✓
K502	S558		✓					
K502	K572			✓				
K502	K730	✓	✓		✓	✓		
K502	K735				✓			
K502	K736			✓	✓			
K502	K758							✓
S503	K730				✓			
S503	K736				✓			
K572	K730	✓			✓			
K724	K735		✓	✓				
K730	K735	✓	✓	✓			✓	✓
K730	K736	✓	✓	✓	✓	✓	✓	✓
K730	K758		✓			✓	✓	✓
K735	K758					✓		✓
K736	K758						✓	

Table S4: Summary of intramolecular cross-links within the bMunc13-2 segment-A monomer, cross-linker BS^3. bMunc13-2 is indicated from the start Met.

Cross-linked amino acid 1	Cross-linked amino acid 2	Calculated molecular weight $[M+H]^+$	Charge state	Peptide 1	Peptide 2	BS^3 D_0/D_4
K380	K386	1994.023	3	$_{376}$DGSAK$_{380}$	$_{381}$QSDVSKLQDEVK$_{392}$	D_4
K473	K502	1618.895	3	$_{473}$KVNAE$_{477}$	$_{497}$DLLADKSR$_{504}$	D_4
K473	K502	1890.023	3	$_{473}$KVNAEDR$_{479}$	$_{497}$DLLADKSR$_{504}$	D_4
K473	K502	2460.288	4	$_{471}$AEKVNAEDR$_{479}$	$_{494}$QLEDLLADKSR$_{504}$	D_4
K473	K572	2162.150	4	$_{471}$AEKVNAEDR$_{479}$	$_{568}$FHTTKLSR$_{575}$	D_4
K473	K736	1571.839	3	$_{471}$AEKVNAEDR$_{479}$	$_{734}$QKK$_{736}$	D_0
K473	K736	1575.864	3, 4	$_{471}$AEKVNAEDR$_{479}$	$_{734}$QKK$_{736}$	D_4
K502	K730	1486.822	3	$_{494}$QLEDLLADKSR$_{504}$	$_{729}$EKR$_{731}$	D_0
K502	K730	1490.847	3	$_{497}$DLLADKSR$_{504}$	$_{729}$EKR$_{731}$	D_4
K502	K730	1861.033	3, 4	$_{494}$QLEDLLADKSR$_{504}$	$_{729}$EKR$_{731}$	D_4
K502	K735	1832.043	4	$_{494}$QLEDLLADKSR$_{504}$	$_{734}$QKK$_{736}$	D_4
K502	K736	1828.017	4	$_{494}$QLEDLLADKSR$_{504}$	$_{734}$QKK$_{736}$	D_0
K502	K736	1832.043	4	$_{494}$QLEDLLADKSR$_{504}$	$_{734}$QKK$_{736}$	D_4
S503	K730	1861.033	3	$_{494}$QLEDLLADKSR$_{504}$	$_{729}$EKR$_{731}$	D_4
S503	K736	1185.695	2	$_{503}$SRR$_{505}$	$_{736}$KLLQE$_{740}$	D_0
S503	K736	1189.720	2	$_{503}$SRR$_{505}$	$_{736}$KLLQE$_{740}$	D_4
S503	K736	1461.857	3	$_{497}$DLLADKSR$_{504}$	$_{734}$QKK736	D_4
K572	K730	1562.895	3	$_{568}$FHTTKLSR$_{575}$	$_{729}$EKR$_{731}$	D_4
K730	K736	1199.699	2, 3	$_{729}$EKR$_{731}$	$_{736}$KLLQE$_{740}$	D_0
K730	K736	1203.725	2, 3	$_{729}$EKR$_{731}$	$_{736}$KLLQE$_{740}$	D_4
K730	K758	2639.430	4	$_{729}$EKR$_{731}$	$_{741}$LVQTASHLSVEDIPSEGKR$_{759}$	D_4
K735	K758	2610.440	4	$_{734}$QKK$_{736}$	$_{741}$LVQTASHLSVEDIPSEGKR$_{759}$	D_4

Table S5: Summary of intramolecular cross-links within the bMunc13-2 segment-A monomer, cross-linker DSBU.bMunc13-2 residues starting with the initial Met; B denotes carbamidomethylated Cys.

Cross-linked amino acid 1	Cross-linked amino acid 2	Calculated molecular weight [M+H]$^+$	Charge state	Peptide 1	Peptide 2
S378	K386	1820.851	3	$_{376}$DGSAK$_{380}$	$_{381}$QSDVSKLQDE$_{390}$
K380	K386	1820.851	3	$_{376}$DGSAK$_{380}$	$_{381}$QSDVSKLQDE$_{390}$
K380	K386	1576.781	2, 3	$_{376}$DGSAKQS$_{382}$	$_{383}$DVSKLQ$_{388}$
K380	K473	1703.819	3	$_{376}$DGSAK$_{380}$	$_{471}$AEKVNAEDR$_{479}$
K380	K473	2148.053	3	$_{376}$DGSAKQSDVSK$_{386}$	$_{473}$KVNAEDR$_{479}$
K380	K473	2348.132	3	$_{376}$DGSAKQSDVSK$_{386}$	$_{471}$AEKVNAEDR$_{479}$
K380	K502	2234.126	3	$_{376}$DGSAKQSDVSK$_{386}$	$_{497}$DLLADKSR$_{504}$
K380	K736	1719.887	3	$_{376}$DGSAKQSDVSK$_{386}$	$_{734}$QKK$_{736}$
S382	K473	2148.053	3	$_{376}$DGSAKQSDVSK$_{386}$	$_{473}$KVNAEDR$_{479}$
K386	K473	2148.053	3	$_{376}$DGSAKQSDVSK$_{386}$	$_{473}$KVNAEDR$_{479}$
K386	K502	1931.008	3	$_{384}$VSKLQDE$_{390}$	$_{497}$DLLADKSR$_{504}$
S418	K473	1924.050	3	$_{416}$GSSPVLIPK$_{424}$	$_{473}$KVNAEDR$_{479}$
K435	K502	1476.817	3	$_{431}$LQIFKQ$_{436}$	$_{501}$DKSR$_{504}$
K442	S451	2315.123	3, 4	$_{441}$HKABNVTK$_{448}$	$_{449}$LQSDBNNAIK$_{458}$
K442	K473	1984.003	3	$_{441}$HKABNVTK$_{448}$	$_{473}$KVNAEDR$_{479}$
K442	K502	2070.076	3	$_{441}$HKABNVTK$_{448}$	$_{497}$DLLADKSR$_{504}$
K448	K473	1984.003	4	$_{441}$HKABNVTK$_{448}$	$_{473}$KVNAEDR$_{479}$
K448	K502	1720.865	3	$_{443}$ABNVTKLQS$_{451}$	$_{501}$DKSR$_{504}$
S466	K473	2175.096	2, 3	$_{459}$ASSBLSLSGPLKAE$_{472}$	$_{473}$KVNAE$_{477}$
K473	K502	1145.627	2	$_{473}$KVNAE$_{477}$	$_{502}$KSR$_{504}$
K473	K502	1672.886	2	$_{473}$KVNAE$_{477}$	$_{497}$DLLADKSR$_{504}$
K473	K502	1872,966	2	$_{471}$AEKVNAE$_{477}$	$_{497}$DLLADKSR$_{504}$
K473	K502	1944.014	3, 4	$_{473}$KVNAEDR$_{479}$	$_{497}$DLLADKSR$_{504}$
K473	K502	2144.094	3	$_{471}$AEKVNAEDR$_{479}$	$_{497}$DLLADKSR$_{504}$
K473	K502	2314.200	3	$_{473}$KVNAEDR$_{479}$	$_{494}$QLEDLLADKSR$_{504}$
K473	K502	2514.279	3	$_{471}$AEKVNAEDR$_{479}$	$_{494}$QLEDLLADKSR$_{504}$
K473	K572	2016.062	3	$_{473}$KVNAEDR$_{479}$	$_{568}$FHTTKLSR$_{575}$
K473	K730	1458.766	3	$_{473}$KVNAEDR$_{479}$	$_{729}$EKR$_{731}$
K473	K730	1658.846	3, 4	$_{471}$AEKVNAEDR$_{479}$	$_{729}$EKR$_{731}$
K473	K735	1629.855	3	$_{471}$AEKVNAEDR$_{479}$	$_{734}$QKK$_{736}$
K473	K736	1385.763	2	$_{473}$KVNAE$_{477}$	$_{736}$KLLQE$_{740}$
K502	S558	2123.015	3	$_{501}$DKSR$_{504}$	$_{449}$DKSSBVLGGSQE$_{460}$
K502	K572	2102,135	4	$_{497}$DLLADKSR$_{504}$	$_{568}$FHTTKLSR$_{575}$
K502	K730	1544.839	3	$_{497}$DLLADKSR$_{504}$	$_{729}$EKR$_{731}$
K502	K730	1700.940	3, 4	$_{497}$DLLADKSRR$_{505}$	$_{729}$EKR$_{731}$
K502	K730	1915.024	3	$_{494}$QLEDLLADKSR$_{504}$	$_{729}$EKR$_{731}$
K502	K736	1742.965	2	$_{497}$DLLADKSR$_{504}$	$_{736}$KLLQE$_{740}$
K572	K730	1616.887	3, 4	$_{568}$FHTTKLSR$_{575}$	$_{729}$EKR$_{731}$
K572	K730	1858.066	3	$_{566}$IKFHTTKLSR$_{575}$	$_{729}$EKR$_{731}$
K724	K735	1876.022	3, 4	$_{719}$BINNFKNVLR$_{728}$	$_{734}$QKK$_{736}$
K730	K735	1299.786	3, 4	$_{729}$EKR$_{731}$	$_{732}$LRQKK$_{736}$
K730	K736	1257.716	2, 3	$_{729}$EKR$_{731}$	$_{736}$KLLQE$_{740}$
K730	K758	1528.808	3	$_{729}$EKR$_{731}$	$_{752}$DIPSEGKR$_{759}$

Table S6: Summary of bMunc13-2 intramolecular cross-links within the CaM/bMunc13-2 segment-A (1:1) complex, cross-linker DSBU. The start methionine is included in the numbers; B denotes carbamidomethylated Cys.

Cross-linked amino acid 1	Cross-linked amino acid 2	Calculated molecular weight $[M+H]^+$	Charge state	Peptide 1	Peptide 2
K380	K386	1820.851	2	$_{376}$DGSAK$_{380}$	$_{381}$QSDVSKLQDE$_{390}$
K380	K386	1576.781	2, 3	$_{376}$DGSAKQS$_{382}$	$_{383}$DVSKLQ$_{388}$
K380	K392	2275.105	3	$_{376}$DGSAKQS$_{382}$	$_{389}$DEVKGTSGAPQVIS$_{402}$
K380	K473	2148.053	3	$_{376}$DGSAKQSDVSK$_{386}$	$_{473}$KVNAEDR$_{479}$
K380	K735	1719.887	3	$_{376}$DGSAKQSDVSK$_{386}$	$_{734}$QKK$_{736}$
K386	K502	1389.733	2, 3	$_{383}$DVSKLQ$_{388}$	$_{501}$DKSR$_{504}$
K392	K502	2088.056	3	$_{389}$DEVKGTSGAPQVIS$_{402}$	$_{501}$DKSR$_{504}$
K442	K473	1984.003	3	$_{441}$HKABNVTK$_{448}$	$_{473}$KVNAEDR$_{479}$
K442	S451	1958.924	3, 4	$_{437}$DSQEHKABNVTK$_{448}$	$_{449}$LQS$_{451}$
K442	K502	2273.105	4	$_{437}$DSQEHKABNVTK$_{448}$	$_{501}$DKSRR$_{505}$
K473	K502	1944.014	3	$_{473}$KVNAEDR$_{479}$	$_{497}$DLLADKSR$_{504}$
K473	K502	1460.734	3	$_{471}$AEKVNAE$_{477}$	$_{501}$DKSR$_{504}$
K473	K730	1458.766	3	$_{473}$KVNAEDR$_{479}$	$_{729}$EKR$_{731}$
K473	K735	1429.776	3	$_{473}$KVNAEDR$_{479}$	$_{734}$QKK$_{736}$
K493	K502	1742.928	2, 3	$_{488}$DILSPKQLE$_{496}$	$_{501}$DKSR$_{504}$
K502	K550	2123.015	3	$_{449}$DKSSBVLGGSQE$_{460}$	$_{501}$DKSRR$_{505}$
K502	K758	1757.925	3	$_{501}$DKSRR$_{505}$	$_{752}$DIPSEGKR$_{759}$
K730	K735	1299.786	2, 3	$_{729}$EKR$_{731}$	$_{732}$LRQKK$_{736}$
K730	K736	1257.716	2	$_{729}$EKR$_{731}$	$_{736}$KLLQE$_{740}$
K730	K736	1299.786	2, 3, 4	$_{729}$EKR$_{731}$	$_{732}$LRQKK$_{736}$
K730	K758	1528.808	2, 3	$_{729}$EKR$_{731}$	$_{752}$DIPSEGKR$_{759}$
K735	K758	1499.818	3	$_{734}$QKK$_{736}$	$_{752}$DIPSEGKR$_{759}$
K736	K758	1499.818	3	$_{734}$QKK$_{736}$	$_{752}$DIPSEGKR$_{759}$

Table S7: Photo-Met cross-links with CaM and bMunc13-2 segment-B at different Ca^{2+} concentrations. x denotes photo-Met.

**Cross-linked amino acids CaM**	**Cross-linked amino acids bMunc13-2**	**Ca^{2+} concentration**	
		750 nM	1 mM
x71/x72	E707	✓	✓
x71/x72	E740	✓	✓
x76	E707	✓	✓
x144/x145	E707	✓	
x144/x145	Y715	✓	✓
x144/x145	C719	✓	✓

Table S8: Summary of intermolecular cross-links between CaM and bMunc13-2 segment-B. x denotes photo-Met, B: carbamidomethylated Cys and m: oxidized Met. The start methionine is not NOT included in the residue numbers of CaM , for bMunc13-2, numbers include the start methionine.

Cross-Linker	Cross-linked amino acids CaM	Cross-linked amino acids bMunc13-2	Calculated molecular weight $[M+H]^+$	Charge state	Amino acid sequence CaM	Amino acid sequence bMunc13-2
Photo-Met	x71/x72	E707	1896.956	3	$_{68}$FLTxMAR$_{74}$ / $_{68}$FLTMxAR$_{74}$	$_{704}$LSLEQVBAE$_{712}$
	x71/x72	E707	1912.951	2, 3	$_{68}$FLTxmAR$_{74}$ / $_{68}$FLTmxAR$_{74}$	$_{704}$LSLEQVBAE$_{712}$
	x71/x72	E740	3257.709	4, 5	$_{68}$FLTxmAR$_{74}$ / $_{68}$FLTmxAR$_{74}$	$_{737}$LLQELVQTASHLSVEDIPSEGK$_{758}$
	x71/x72	E740	3413.810	4, 5	$_{68}$FLTxmAR$_{74}$ / $_{68}$FLTmxAR$_{74}$	$_{737}$LLQELVQTASHLSVEDIPSEGKR$_{759}$
	x71/x72	E740	3526.857	5	$_{68}$FLTxmAR$_{74}$ / $_{68}$FLTmxAR$_{74}$	$_{737}$LLQELVQTASHLSVEDIPSEGKRE$_{760}$
	x76	E707	1433.767	2, 3	$_{75}$KxK$_{77}$	$_{704}$LSLEQVBAE$_{712}$
	x76	E707	2166.184	4	$_{75}$KxK$_{77}$	$_{704}$LSLEQVBAETIYLNK$_{718}$
	x76	E707	2239.028	3, 4	$_{75}$KxKDTDSEEE$_{84}$	$_{704}$LSLEQVBAE$_{712}$
	x76	E707	2380.118	3	$_{76}$xKDTDSEEEIR$_{86}$	$_{704}$LSLEQVBAE$_{712}$
	x76	E707	2508.213	4	$_{75}$KxKDTDSEEEIR$_{86}$	$_{704}$LSLEQVBAE$_{712}$
	x76	E707	2509.161	3, 4	$_{76}$xKDTDSEEEIRE$_{87}$	$_{704}$LSLEQVBAE$_{712}$
	x76	E707	2637.256	4, 5	$_{75}$KxKDTDSEEEIRE$_{87}$	$_{704}$LSLEQVBAE$_{712}$
	x144/x145	E707	2225.119	3	$_{141}$FVQMxTAKLE$_{150}$ / $_{141}$FVQMxTAKLE$_{150}$	$_{704}$LSLEQVBAE$_{712}$
	X144/x145	Y715	1701.924	3, 4	$_{141}$FVQxmTAK$_{148}$ / $_{141}$FVQmxTAK$_{148}$	$_{713}$TIYLNK$_{718}$
	x144/x145	C719	1672.855	3, 4	$_{141}$FVQxMTAK$_{148}$ / $_{141}$FVQMxTAK$_{148}$	$_{719}$CINNFK$_{724}$
	x144/x145	C719	1688.850	3, 4	$_{141}$FVQxmTAK$_{148}$ / $_{141}$FVQmxTAK$_{148}$	$_{719}$CINNFK$_{724}$

Table S9: Photo-Met cross-links with CaM and bMunc13-2 segment-C at different Ca^{2+} concentrations. x denotes photo-Met.

Cross-linked amino acids CaM	Cross-linked amino acids bMunc13-2	Ca^{2+} concentration					
		750 nM	2.5 µM	7.5 µM	100 µM	300 µM	1 mM
x36	E712	✓	✓	✓	✓		✓
x71/x72	E707	✓	✓	✓	✓	✓	✓
x71/x72	E729				✓	✓	
x76	E707	✓	✓	✓	✓	✓	✓
x76	E729				✓		
x124	E729						✓
x144/x145	E707			✓			
x144/x145	Y715			✓			✓
x144/x145	C719	✓	✓		✓	✓	✓
x144/x145	E729	✓	✓	✓	✓	✓	

Table S10: Summary of intermolecular cross-links between CaM and bMunc13-2 segment-C. x denotes photo-Met, B: carbamidomethylated Cys and m: oxidized Met. CaM without the initial Met, bMunc13-2 numbers include the initial Met; segment-C is *N*-terminally acetylated and *C*-terminally amidated.

Cross-Linker	Cross-linked amino acids CaM	Cross-linked amino acids bMunc13-2	Calculated molecular weight [M+H]$^+$	Charge state	Amino acid sequence CaM	Amino acid sequence bMunc13-2
Photo-Met	x36	E707	1874.010	3	$_{32}$LGTVxR$_{37}$	$_{703}$KLSLEQVBAE$_{712}$
	x36	E712	1994.074	3	$_{32}$LGTVxR$_{37}$	$_{708}$QVBAETIYLNK$_{718}$
	x36	E712	2123.117	4	$_{31}$ELGTVxR$_{37}$	$_{708}$QVBAETIYLNK$_{718}$
	x36	E712	2735.465	4	$_{31}$ELGTVxR$_{37}$	$_{703}$KLSLEQVBAETIYLNK$_{718}$
	x71/x72	E707	1896.956	3	$_{68}$FLTxMAR$_{74}$ / $_{68}$FLTMxAR$_{74}$	$_{704}$LSLEQVBAE$_{712}$
	x71/x72	E707	1912.951	3	$_{68}$FLTxmAR$_{74}$ / $_{68}$FLTmxAR$_{74}$	$_{704}$LSLEQVBAE$_{712}$
	x71/x72	E707	2067.061	3	$_{68}$FLTxMAR$_{74}$ / $_{68}$FLTMxAR$_{74}$	$_{703}$KLSLEQVBAE$_{712}$
	x71/x72	E707	2083.056	3	$_{68}$FLTxmAR$_{74}$ / $_{68}$FLTmxAR$_{74}$	$_{703}$KLSLEQVBAE$_{712}$
	x71/x72	E729	1296.709	3	$_{68}$FLTxmAR$_{74}$ / $_{68}$FLTmxAR$_{74}$	$_{729}$EKR$_{731}$
	x76	E707	1821.896	4	$_{75}$KxKDTDSEEE$_{84}$	$_{703}$KLSLE$_{707}$
	x76	E707	2220.124	4	$_{75}$KxKDTDSEEEIRE$_{87}$	$_{703}$KLSLE$_{707}$
	x76	E707	2281.039	3	$_{76}$xKDTDSEEE$_{84}$	$_{703}$KLSLEQVBAE$_{712}$
	x76	E707	2380.118	3	$_{76}$xKDTDSEEEIR$_{86}$	$_{704}$LSLEQVBAE$_{712}$
	x76	E707	2409.134	3, 4	$_{75}$KxKDTDSEEE$_{84}$	$_{703}$KLSLEQVBAE$_{712}$
	x76	E707	2508.213	4	$_{75}$KxKDTDSEEEIR$_{86}$	$_{704}$LSLEQVBAE$_{712}$
	x76	E707	2509.161	3, 4	$_{76}$xKDTDSEEEIRE$_{87}$	$_{704}$LSLEQVBAE$_{712}$
	x76	E707	2550.224	4	$_{76}$xKDTDSEEEIR$_{86}$	$_{703}$KLSLEQVBAE$_{712}$
	x76	E707	2637.256	4	$_{75}$KxKDTDSEEEIRE$_{87}$	$_{704}$LSLEQVBAE$_{712}$
	x76	E707	2678.319	4	$_{75}$KxKDTDSEEEIR$_{86}$	$_{703}$KLSLEQVBAE$_{712}$
	x76	E707	2679.267	4	$_{76}$xKDTDSEEEIRE$_{87}$	$_{703}$KLSLEQVBAE$_{712}$
	x76	E707	2807.362	4, 5	$_{75}$KxKDTDSEEEIRE$_{87}$	$_{703}$KLSLEQVBAE$_{712}$
	x76	E729	2021.015	3	$_{75}$KxKDTDSEEEIRE$_{87}$	$_{729}$EKR$_{731}$
	x124	E729	1760.902	3, 4	$_{116}$LTDEEVDExIR$_{126}$	$_{729}$EKR$_{731}$
	x124	E729	1889.945	4	$_{116}$LTDEEVDExIRE$_{127}$	$_{729}$EKR$_{731}$
	x144/x145	E707	1982.993	3	$_{141}$FVQxMTAK$_{148}$ / $_{141}$FVQMxTAK$_{148}$	$_{704}$LSLEQVBAE$_{712}$
	X144/x145	Y715	1685.929	3	$_{141}$FVQxMTAK$_{148}$ / $_{141}$FVQMxTAK$_{148}$	$_{713}$TIYLNK$_{718}$
	X144/x145	Y715	1701.924	3, 4	$_{141}$FVQxmTAK$_{148}$ / $_{141}$FVQmxTAK$_{148}$	$_{713}$TIYLNK$_{718}$
	x144/x145	C719	1672.855	3, 4	$_{141}$FVQxMTAK$_{148}$ / $_{141}$FVQMxTAK$_{148}$	$_{719}$CINNFK$_{724}$
	x144/x145	C719	1688.850	3, 4	$_{141}$FVQxmTAK$_{148}$ / $_{141}$FVQmxTAK$_{148}$	$_{719}$CINNFK$_{724}$
	x144/x145	C719	3223.456	4	$_{126}$EADIDGDGQVNYEEFVQxmTAK$_{148}$ / $_{126}$EADIDGDGQVNYEEFVQmxTAK$_{148}$	$_{719}$CINNFK$_{724}$
	x144/x145	E729	1366.751	3, 4	$_{141}$FVQxMTAK$_{148}$ / $_{141}$FVQMxTAK$_{148}$	$_{729}$EKR$_{731}$
	x144/x145	E729	1624.873	3	$_{141}$FVQxmTAKLE$_{150}$ / $_{141}$FVQmxTAKLE$_{150}$	$_{729}$EKR$_{731}$

Table S11: Summary of intermolecular cross-links between CaM and bMunc13-2 segment-A-V709D at 750 nM Ca^{2+}. x denotes photo-Met, B: carbamidomethylated Cys and m: oxidized Met. CaM without the initial Met, bMunc13-2 with the initial Met.

Cross-Linker	Cross-linked amino acids CaM	Cross-linked amino acids bMunc13-2	Calculated molecular weight $[M+H]^+$	Charge state	Amino acid sequence CaM	Amino acid sequence bMunc13-2
Photo-Met	x36	D549	1277.721	3	$_{32}$LGTVxR$_{37}$	$_{446}$NVFDK$_{450}$
	x36	E565	1258.737	3	$_{32}$LGTVxR$_{37}$	$_{463}$DVEIK$_{467}$
	x36	E707	1719.858	2	$_{32}$LGTVxR$_{37}$	$_{704}$LSLEQDBAE$_{712}$
	x36	D709	1406.658	2	$_{31}$ELGTVxR$_{37}$	$_{708}$QDBAE$_{712}$
	x36	D709	1719.858	3	$_{32}$LGTVxR$_{37}$	$_{704}$LSLEQDBAE$_{712}$
	x36	D709	1848.901	2, 3	$_{31}$ELGTVxR$_{37}$	$_{704}$LSLEQDBAE$_{712}$
	x51	E612	1907.858	3	$_{48}$LQDxINE$_{54}$	$_{607}$SGSQSENSDR$_{616}$
	x51	D709	1463.632	2	$_{48}$LQDxINE$_{54}$	$_{708}$QDBAE$_{712}$
	x71/x72	E496	1893.015	4	$_{68}$FLTxMAR$_{74}$ / $_{68}$FLTMxAR$_{74}$	$_{494}$QLEDLLADK$_{502}$
	x71/x72	E707	1855.893	3	$_{68}$FLTxMAR$_{74}$ / $_{68}$FLTMxAR$_{74}$	$_{704}$LSLEQDCAE$_{712}$
	x71/x72	E707	1912.914	2, 3	$_{68}$FLTxMAR$_{74}$ / $_{68}$FLTMxAR$_{74}$	$_{704}$LSLEQDBAE$_{712}$
	x71/x72	E707	1928.909	3	$_{68}$FLTxmAR$_{74}$ / $_{68}$FLTmxAR$_{74}$	$_{704}$LSLEQDBAE$_{712}$
	x76	E707	2254.987	3	$_{75}$KxKDTDSEEE$_{84}$	$_{704}$LSLEQDBAE$_{712}$
	x76	E707	2524.172	4	$_{75}$KxKDTDSEEEIR$_{86}$	$_{704}$LSLEQDBAE$_{712}$
	x76	E707	2653.215	4, 5	$_{75}$KxKDTDSEEEIRE$_{87}$	$_{704}$LSLEQDBAE$_{712}$
	x76	E729	2021.015	4, 5	$_{75}$KxKDTDSEEEIRE$_{87}$	$_{729}$EKR$_{731}$
	x76	H578/H579	1970.957	4	$_{75}$KxKDTDSEEE$_{84}$	$_{576}$AIHHFR$_{561}$
	x76	H578/H579	2369.184	5	$_{75}$KxKDTDSEEEIRE$_{87}$	$_{576}$AIHHFR$_{561}$
	x109	D709	2071.996	3	$_{107}$HVxTNLGEK$_{113}$	$_{704}$LSLEQDBAE$_{712}$
	x124	D709	2393.102	3	$_{116}$LTDEEVDExIR$_{126}$	$_{704}$LSLEQDBAE$_{712}$
	x124	D709	2522.145	3	$_{116}$LTDEEVDExIRE$_{127}$	$_{704}$LSLEQDBAE$_{712}$
	x144/x145	C719	1688.850	4	$_{141}$FVQxmTAK$_{148}$ / $_{141}$FVQmxTAK$_{148}$	$_{719}$CINNFK$_{724}$
	x144/x145	E707	1941.930	3	$_{141}$FVQxMTAK$_{148}$ / $_{141}$FVQMxTAK$_{148}$	$_{704}$LSLEQDCAE$_{712}$
	x144/x145	E707	1998.951	3	$_{141}$FVQxMTAK$_{148}$ / $_{141}$FVQMxTAK$_{148}$	$_{704}$LSLEQDBAE$_{712}$
	x144/x145	E707	2014.946	3	$_{141}$FVQxmTAK$_{148}$ / $_{141}$FVQmxTAK$_{148}$	$_{704}$LSLEQDBAE$_{712}$
	x144/x145	E729	1366.751	3, 4	$_{141}$FVQxMTAK$_{148}$ / $_{141}$FVQMxTAK$_{148}$	$_{729}$EKR$_{731}$

Table S12: Summary of intermolecular cross-links between CaM and bMunc13-2 segment-A-I714D at 750 nM Ca^{2+}. x denotes photo-Met, B: carbamidomethylated Cys and m: oxidized Met. CaM without bMunc13-2 with initial Met.

Cross-Linker	Cross-linked amino acids CaM	Cross-linked amino acids bMunc13-2	Calculated molecular weight $[M+H]^+$	Charge state	Amino acid sequence CaM	Amino acid sequence bMunc13-2
Photo-Met	x36	D452	1760,932	3	$_{32}$LGTVxR$_{37}$	$_{449}$LQSDBNNAIK$_{458}$
	x36	D452	1817,954	3	$_{32}$LGTVxR$_{37}$	$_{449}$LQSDBNNAIK$_{458}$
	x36	D549	1277,721	3	$_{32}$LGTVxR$_{37}$	$_{446}$NVFDK$_{450}$
	x36	E565	1258,737	2, 3, 4	$_{32}$LGTVxR$_{37}$	$_{463}$DVEIK$_{467}$
	x36	E712	1996.017	3, 4	$_{32}$LGTVxR$_{37}$	$_{708}$QVBAETDYLNK$_{718}$
	x51	D549	1463.738	3	$_{48}$LQDxINE$_{54}$	$_{446}$NVFDK$_{450}$
	x51	E565	1444,753	3	$_{48}$LQDxINE$_{54}$	$_{463}$DVEIK$_{467}$
	x51	H579	1621,845	3	$_{48}$LQDxINE$_{54}$	$_{576}$AIHHFR$_{561}$
	x51	E603	2015.940	2	$_{48}$LQDxINE$_{54}$	$_{503}$NNGSISPEDLE$_{603}$
	x51	E612	1907,858	3	$_{48}$LQDxINE$_{54}$	$_{607}$SGSQSENSDR$_{616}$
	x51	D697	2188.030	3, 4	$_{48}$LQDxINE$_{54}$	$_{692}$TBSRPDSPNQGK$_{703}$
	x51	E712	2182.033	3	$_{48}$LQDxINE$_{54}$	$_{708}$QVBAETDYLNK$_{718}$
	x71/x72	E496	1893,015	3	$_{68}$FLTxMAR$_{74}$ / $_{68}$FLTMxAR$_{74}$	$_{494}$QLEDLLADK$_{502}$
	x71/x72	D549	1470,777	3	$_{68}$FLTxMAR$_{74}$ / $_{68}$FLTMxAR$_{74}$	$_{446}$NVFDK$_{450}$
	x71/x72	E565	1451,793	3, 4	$_{68}$FLTxMAR$_{74}$ / $_{68}$FLTMxAR$_{74}$	$_{463}$DVEIK$_{467}$
	x71/x72	H578	1628,884	3, 4	$_{68}$FLTxMAR$_{74}$ / $_{68}$FLTMxAR$_{74}$	$_{576}$AIHHFR$_{561}$
	x71/x72	E707	1896.956	3	$_{68}$FLTxMAR$_{74}$ / $_{68}$FLTMxAR$_{74}$	$_{704}$LSLEQVBAE$_{712}$
	x71/x72	E707	1912.951	3	$_{68}$FLTxmAR$_{74}$ / $_{68}$FLTmxAR$_{74}$	$_{704}$LSLEQVBAE$_{712}$
	x71/x72	C719	1586,818	3	$_{68}$FLTxMAR$_{74}$ / $_{68}$FLTMxAR$_{74}$	[CINNFK]
	x76	E707	2182,007	3	$_{75}$KxKDTDSEEE$_{84}$	$_{704}$LSLEQVCAE$_{712}$
	x76	E707	2509.161	4	$_{76}$xKDTDSEEEIRE$_{87}$	$_{704}$LSLEQVBAE$_{712}$
	x76	E707	2637.256	4	$_{75}$KxKDTDSEEEIRE$_{87}$	$_{704}$LSLEQVBAE$_{712}$
	x124	E516/E517	2750.289	3	$_{116}$LTDEEVDExIRE$_{127}$	$_{506}$FATLNPDSAVEE$_{517}$
	x144/x145	E562	2545.143	3	$_{141}$FVQxMTAK$_{148}$ / $_{141}$FVQMxTAK$_{148}$	$_{451}$SSBVLGGSQEDEDVE$_{465}$
	x144/x145	E565	1537,829	3	$_{141}$FVQxMTAK$_{148}$ / $_{141}$FVQMxTAK$_{148}$	$_{463}$DVEIK$_{467}$
	x144/x145	H578	1714,921	3	$_{141}$FVQxMTAK$_{148}$ / $_{141}$FVQMxTAK$_{148}$	$_{576}$AIHHFR$_{561}$
	x144/x145	E707	1925,971	3	$_{141}$FVQxMTAK$_{148}$ / $_{141}$FVQMxTAK$_{148}$	$_{704}$LSLEQVCAE$_{712}$
	x144/x145	E707	1982,993	3	$_{141}$FVQxMTAK$_{148}$ / $_{141}$FVQMxTAK$_{148}$	$_{704}$LSLEQVBAE$_{712}$
	x144/x145	D714	1687,872	3	$_{141}$FVQxMTAK$_{148}$ / $_{141}$FVQMxTAK$_{148}$	$_{713}$TDYLNK$_{718}$
	x144/x145	C719	1672,855	3	$_{141}$FVQxMTAK$_{148}$ / $_{141}$FVQMxTAK$_{148}$	$_{719}$CINNFK$_{724}$
	x144/x145	C719	1688.850	4	$_{141}$FVQxmTAK$_{148}$ / $_{141}$FVQmxTAK$_{148}$	$_{719}$CINNFK$_{724}$
	x144/x145	E729	1366.751	4	$_{141}$FVQxMTAK$_{148}$ / $_{141}$FVQMxTAK$_{148}$	$_{729}$EKR$_{731}$

Table S13: Summary of intermolecular cross-links between CaM and bMunc13-2 segment-A-F723D at 750 nM Ca^{2+}. x denotes photo-Met, B: carbamidomethylated Cys and m: oxidized Met. CaM without the initial Met, bMunc13-2 the initial Met.

Cross-Linker	Cross-linked amino acids CaM	Cross-linked amino acids bMunc13-2	Calculated molecular weight $[M+H]^+$	Charge state	Amino acid sequence CaM	Amino acid sequence bMunc13-2
Photo-Met	x36	D452	1817.954	3, 4	$_{32}$LGTVxR$_{37}$	$_{449}$LQSDBNNAIK$_{458}$
	x36	D478	1358.739	3	$_{32}$LGTVxR$_{37}$	$_{472}$VNAEDR$_{479}$
	x36	D549	1277.721	3	$_{32}$LGTVxR$_{37}$	$_{446}$NVFDK$_{450}$
	x36	E565	1258.737	3, 4	$_{32}$LGTVxR$_{37}$	$_{463}$DVEIK$_{467}$
	x36	E565	2507.229	4	$_{32}$LGTVxR$_{37}$	$_{451}$SSBVLGGSQEDEDVEIK$_{467}$
	x51	E603	2015.940	2	$_{48}$LQDxINE$_{54}$	$_{503}$NNGSISPEDLE$_{603}$
	x51	D697	2188.030	3, 4	$_{48}$LQDxINE$_{54}$	$_{692}$TBSRPDSPNQGK$_{703}$
	x71/x72	E496	1893.015	4	$_{68}$FLTxMAR$_{74}$ / $_{68}$FLTMxAR$_{74}$	$_{494}$QLEDLLADK$_{502}$
	x71/x72	D549	1470.777	3	$_{68}$FLTxMAR$_{74}$ / $_{68}$FLTMxAR$_{74}$	$_{446}$NVFDK$_{450}$
	x71/x72	E707	1839.934	3	$_{68}$FLTxMAR$_{74}$ / $_{68}$FLTMxAR$_{74}$	$_{704}$LSLEQVBAE$_{712}$
	x71/x72	E707	1896.956	3	$_{68}$FLTxMAR$_{74}$ / $_{68}$FLTMxAR$_{74}$	$_{704}$LSLEQVBAE$_{712}$
	x71/x72	H578/H579	1628.884	4	$_{68}$FLTxMAR$_{74}$ / $_{68}$FLTMxAR$_{74}$	$_{576}$AIHHFR$_{561}$
	x76	H578/H579	1970.957	4	$_{75}$KxKDTDSEEE$_{84}$	$_{576}$AIHHFR$_{561}$
	x76	H578/H579	2369.184	4, 5	$_{75}$KxKDTDSEEEIRE$_{87}$	$_{576}$AIHHFR$_{561}$
	x76	E612	2655.198	4	$_{75}$KxKDTDSEEEIRE$_{87}$	$_{607}$SGSQSENSDR$_{616}$
	x76	E707	2050.018	3	$_{75}$KxKDTDSEEEIRE$_{87}$	$_{704}$LSLE$_{707}$
	x76	E707	2239.028	3	$_{75}$KxKDTDSEEE$_{84}$	$_{704}$LSLEQVBAE$_{712}$
	x76	E707	2509.161	3	$_{76}$xKDTDSEEEIRE$_{87}$	$_{704}$LSLEQVBAE$_{712}$
	x76	E707	2580.235	3	$_{75}$KxKDTDSEEEIRE$_{87}$	$_{704}$LSLEQVBAE$_{712}$
	x76	E707	2637.256	3, 4	$_{75}$KxKDTDSEEEIRE$_{87}$	$_{704}$LSLEQVBAE$_{712}$
	x144/x145	E562	2545.143	3	$_{141}$FVQxMTAK$_{148}$ / $_{141}$FVQMxTAK$_{148}$	$_{451}$SSBVLGGSQEDEDVE$_{465}$
	x144/x145	E565	1537.829	3	$_{141}$FVQxMTAK$_{148}$ / $_{141}$FVQMxTAK$_{148}$	$_{463}$DVEIK$_{467}$
	x144/x145	H578/H579	1714.921	4	$_{141}$FVQxMTAK$_{148}$ / $_{141}$FVQMxTAK$_{148}$	$_{576}$AIHHFR$_{561}$
	x144/x145	D615	1425.715	3, 4	$_{141}$FVQxMTAK$_{148}$ / $_{141}$FVQMxTAK$_{148}$	$_{613}$NSDR$_{616}$
	x144/x145	C719	1640.813	3	$_{141}$FVQxMTAK$_{148}$ / $_{141}$FVQMxTAK$_{148}$	$_{719}$CINNDK$_{724}$
	x144/x145	C719	2123.110	4, 5	$_{141}$FVQxMTAK$_{148}$ / $_{141}$FVQMxTAK$_{148}$	$_{719}$CINNDKNVLR$_{728}$
	x144/x145	D723	1697.835	4	$_{141}$FVQxMTAK$_{148}$ / $_{141}$FVQMxTAK$_{148}$	$_{719}$BINNDK$_{724}$
	x144/x145	E729	1366.751	3, 4	$_{141}$FVQxMTAK$_{148}$ / $_{141}$FVQMxTAK$_{148}$	$_{729}$EKR$_{731}$
